# Reactive oxygen species rescue regeneration after silencing the MAPK–ERK signaling pathway in *Schmidtea mediterranea*

**DOI:** 10.1038/s41598-020-79588-1

**Published:** 2021-01-13

**Authors:** V. Jaenen, S. Fraguas, K. Bijnens, M. Heleven, T. Artois, R. Romero, K. Smeets, F. Cebrià

**Affiliations:** 1 Centre for Environmental Sciences, Hasselt University, Diepenbeek, Belgium; 2 Department of Genetics, Microbiology and Statistics, Faculty of Biology, University of Barcelona, Av. Diagonal 643, 08028 Barcelona, Spain; 3 Institute of Biomedicine of the University of Barcelona (IBUB), Barcelona, Spain; 4 Department of Biology and Geology, Faculty of Sciences, Agoralaan Building D, 3590 Diepenbeek, Belgium

**Keywords:** Cell biology, Developmental biology

## Abstract

Despite extensive research on molecular pathways controlling the process of regeneration in model organisms, little is known about the actual initiation signals necessary to induce regeneration. Recently, the activation of ERK signaling has been shown to be required to initiate regeneration in planarians. However, how ERK signaling is activated remains unknown. Reactive Oxygen Species (ROS) are well-known early signals necessary for regeneration in several models, including planarians. Still, the probable interplay between ROS and MAPK/ERK has not yet been described. Here, by interfering with major mediators (ROS, EGFR and MAPK/ERK), we were able to identify wound-induced ROS, and specifically H_2_O_2_, as upstream cues in the activation of regeneration. Our data demonstrate new relationships between regeneration-related ROS production and MAPK/ERK activation at the earliest regeneration stages, as well as the involvement of the EGFR-signaling pathway. Our results suggest that (1) ROS and/or H_2_O_2_ have the potential to rescue regeneration after MEK-inhibition, either by H_2_O_2_-treatment or light therapy, (2) ROS and/or H_2_O_2_ are required for the activation of MAPK/ERK signaling pathway, (3) the EGFR pathway can mediate ROS production and the activation of MAPK/ERK during planarian regeneration.

## Introduction

Regeneration is the fascinating phenomenon in which animals repair and regrow lost or damaged tissues, structures or even the whole body^1^. At the cellular level, this includes processes such as proliferation, migration and differentiation; all of them needed to be under a strict genetic and molecular control^2^. The capacity to regenerate is widely distributed across the animal kingdom^3^. However, the level of regeneration varies significantly among different species; from full-body regeneration in invertebrates such as *Hydra* and planarians to regeneration of specific organs and structures such as limbs in amphibians and the heart in zebrafish^4–6^. Mammals such as humans, on the other hand, have minimal regenerative capabilities. Understanding how tissue development takes place in model organisms might provide fundamental knowledge for the further improvement of regenerative medicine.

In this context, freshwater planarians are a model with several attractive features: (1) they can regenerate a whole body from a tiny piece, (2) a large part of their cells consists of a population of adult pluripotent stem cells called neoblasts, with a similar transcriptional profile as compared with other invertebrate and vertebrate stem cells^7,8^ and (3) they use conserved signaling pathways to regulate cell differentiation, patterning and morphogenesis. Thus, Hedgehog and Wnt/β-catenin signaling are required to re-establish the antero-posterior axis of the animal, the BMP pathway regulates the dorsoventral axis, and the EGFR pathway is needed for proper neoblast differentiation^9–13^. Although several studies have uncovered a pivotal role of these, and other, signaling pathways during planarian regeneration, little is known about which upstream signals activate them in order to trigger a regenerative answer after amputation. Recently, Owlarn and colleagues reported that a planarian extracellular signal-regulated protein kinase (ERK) is strongly activated in a stem cell-independent manner, just minutes after amputation^14^. By inhibiting protein synthesis using cycloheximide, they showed that ERK activation is triggered by injury signals that do not originate from newly synthesized proteins. Therefore, ERK activation by a still unknown factor appears to be the most upstream initiator of planarian regeneration^14^. In recent years, many in vitro as well as in vivo studies put forward reactive oxygen species (ROS) as upstream signaling molecules of regeneration^15–17^. H_2_O_2_, for example, can cross cell membranes through aquaporin channels and gap-junctions and diffuse freely between the cells^18,19^. Similar to what happens in other physiological processes such as growth, inflammation and ageing, wound-induced ROS, and especially the non-radical ROS, H_2_O_2_, with its rather long half-life, can play a regulating role in early wound responses and regeneration. In 2013, Love et al*.* demonstrated that amputation-induced ROS production is required for tadpole tail regeneration in *Xenopus*^20^. Shortly after, Gauron and colleagues showed a similar ROS production at the amputation site during fin regeneration in adult zebrafish and proved its role in blastema formation^21^. More recently, the Serras laboratory demonstrated the presence of an oxidative burst just minutes after inducing regeneration of the wing imaginal disc in *Drosophila*^22^. Also, in planarians, we reported that an amputation-induced ROS burst is necessary for proper stem cell differentiation and successful regeneration^17^.

Many studies have already shown an interplay between ROS and mitogen-activated protein kinases (MAPK) signaling pathways^23,24^. Ruffels et al. simulated a ROS burst by direct exposure of exogenous H_2_O_2_ to human neuroblastoma cells, resulting in a significant increase in ERK activation^25^. In *Drosophila*, ROS-dependent stimulation of MAPKs is essential for the activation of JAK/STAT signaling, which drives regeneration^22^. In planarians^26^ and other models^27–29^, the activation of MAPK pathways can be mediated by the upstream epidermal growth factor receptor (EGFR), and also the activation of the EGFR pathway by ROS has been described in different models^30,31^. Overall, these studies suggest that wound-induced ROS signaling may operate through EGFR–MAPK pathways, stimulating transcriptional expression of regeneration-related genes, crucial for tissue repair and restoration of homeostasis.

Even though ROS, EGFR and ERK are required for planarian regeneration, the relationship between these pathways is not known. Here, we show that amputation-induced ROS production might be the upstream cue that activates ERK signaling to initiate regeneration.

## Results

### Generation of reactive oxygen species after applying an R- or H-wound

**Figure 1:**
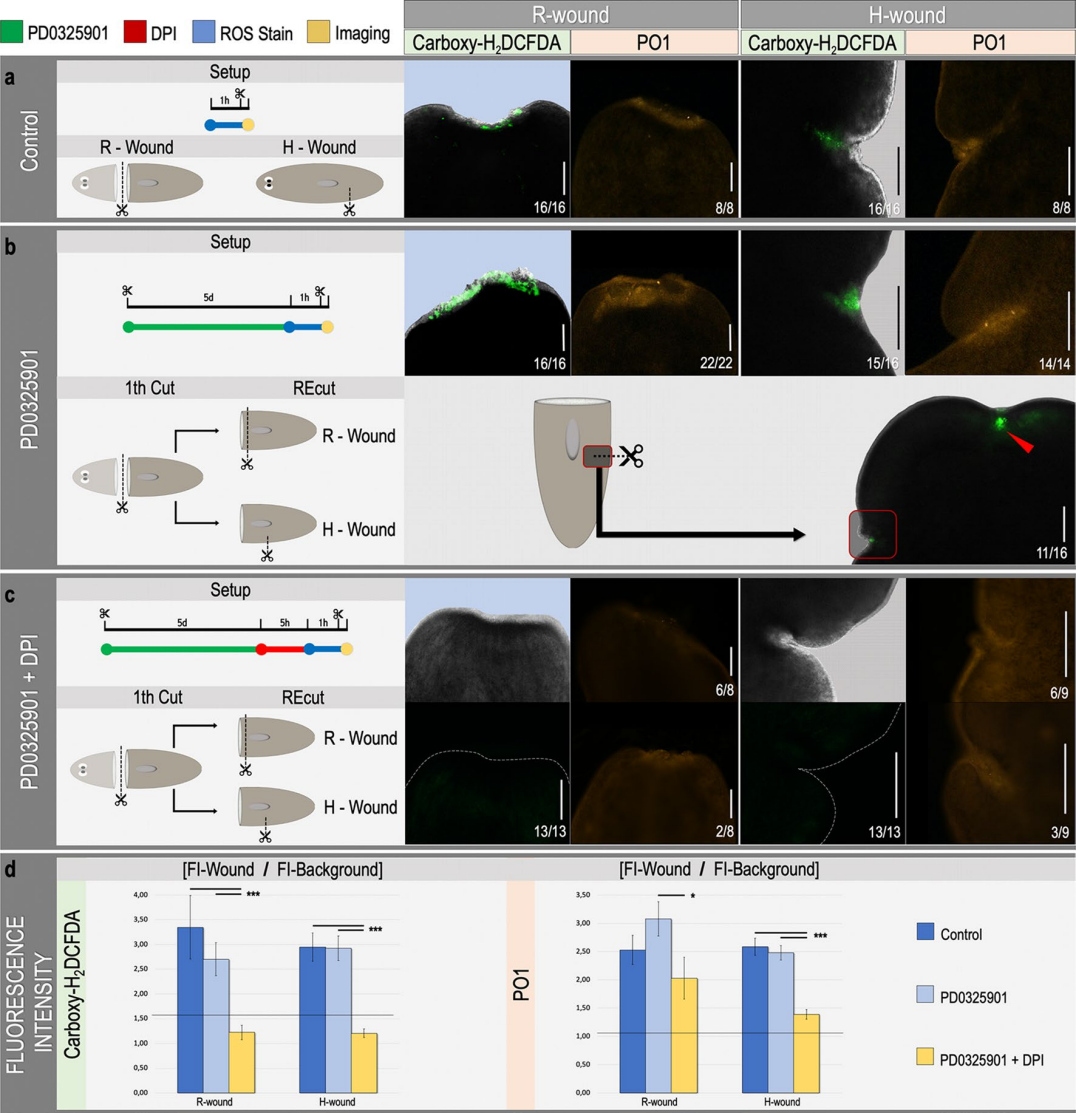
In vivo visualization of ROS production at the amputation site after re-wounding MEK-inhibited dormant fragments. For each condition, the experimental setup is displayed in the left panel. Color code as follows; green: MEK inhibition by PD0325901 (10 μM, 5 days), red: inhibition of ROS production by DPI (3 μM, 5 h), blue: ROS visualization procedure using carboxy-H_2_DCFDA as general ROS indicator or peroxy-orange-1 to specifically stain H_2_O_2_, yellow: imaging. Regenerative wound (R-wound) and healing wound (H-wound). All animals were imaged 30 min post amputation (MPA). A representative close-up merged image of bright field and fluorescence of either R-wounds or H-wounds after the general ROS stain is displayed on the left panel (green fluorescence), and a close-up image of the specific H_2_O_2_ stain is displayed on the right (orange fluorescence) (**a**) ROS production at the site of an R-wound and an H-wound in controls. (**b**) ROS production at the amputation site of an R-wound and an H-wound in MEK-inhibited (PD0325901), dormant fragments. Lower panel in (b) shows an image of the site of the original R-wound applied before MEK-inhibition (red arrowhead) together with the newly applied H-wound (red square). (**c**) ROS production after re-wounding (R-wound and H-wound) MEK-inhibited and DPI exposed dormant fragments. Because of the strongly reduced fluorescence in (c), the close-up of the wound site is shown in bright field (upper left panels) and fluorescence (lower left panels) separated for in the general ROS stain while the H_2_O_2_ stain allows to only display the fluorescence images (on the right panels). The dotted, white lines indicate the border of the wound site. Scale bars 100 µm. (**d**) Relative fluorescence intensity of the wound region. Color code as follows; dark blue: control (representing data of panel A), light blue: MEK inhibition (representing data of panel B), yellow: MEK inhibition + DPI (representing data of panel c). Left panel; fluorescence intensity representing general ROS production visualized with carboxy-H_2_DCFDA. Right panel; fluorescence intensity representing hydrogen peroxide production visualized with peroxy orange 1 (PO1). Statistical significance is indicated by: *p < 0.05; **p < 0.01; ***p < 0.001.

In planarians, a common generic wound response program is triggered by either injuries that require only wound healing (H-wounds) or by injuries that imply tissue loss and therefore require regeneration (R-wounds)^14^. We previously showed an amputation-induced ROS burst after inflicting an R-wound in planarians plus regenerative defects when inhibiting this ROS burst by DPI treatment^17^. Here we confirm the impaired regeneration after inhibition of ROS production (Supplementary Fig. S1) as well as the fast ROS production at the R-wound site, and additionally show the ROS burst after inflicting an H-wound (Fig. 1a, Carboxy-H_2_DCFDA, green signal). Furthermore, because carboxy-H_2_DCFDA is used to stain all intracellular ROS, in which a distinction between the different species cannot be made, we additionally visualized H_2_O_2_ specifically by the aid of PO1 (Peroxy Orange 1). A clear fluorescent signal at both R- and H-wound sites (Fig. 1a, PO1, orange signal) is observed, indicating a significant production of H_2_O_2_ after inflicting a wound.

Owlarn and colleagues showed that MEK inhibition blocks regeneration. These MEK-inhibited fragments remain in a dormant non-regenerative state unless novel R- or H-wounds are inflicted in the dormant wound site. Both wound types trigger generic initiation signals, resulting in the re-activation of the regeneration program^14^. Furthermore, we demonstrated that not only a H-wound inflicted in the dormant wound site, but also more distant wounds were able to rescue regeneration in MEK-inhibited tails at very similar levels based on blastema size and eye development. Moreover, and in accordance with the results of Owlarn et al., the level of rescue was proportional with the amount of wounding, (Supplementary Fig. S2c). These results suggest that the generically induced initiation signals could possibly communicate with the dormant blastema in order to re-initiate regeneration.

In order to check if these initiation signals could possibly be ROS/H_2_O_2_, we first verified their presence at the wound site directly after re-wounding MEK-inhibited fragments. Both, general ROS and H_2_O_2_ were detected at the site of the newly inflicted R- and H-wounds in the MEK-inhibited fragments (Fig. 1b, upper panels). After inflicting an H-wound, ROS levels did not only increase at the H-wound site, but also occurred at the original, R-wound site (Fig. 1b, lower panel, red arrowhead). Treatment with DPI (Diphenyleneiodonium; a nonspecific flavoprotein inhibitor interfering with many different electron transporters, widely used as ROS inhibitor) resulted in a robust decrease of ROS/H_2_O_2_-signals at all wound sites (Fig. 1c). Quantification of the fluorescence intensity confirmed the statistically significant decrease in ROS production (general ROS and H_2_O_2_) at the newly inflicted R- or H-wound in MEK-inhibited tails after treatment with DPI (Fig. 1d). Correspondingly, a significant decrease in regeneration was observed when combining a new R-wound in MEK-inhibited tails with treatment with DPI (Supplementary Fig. S2b). Negative controls (without carboxy-H_2_DCFDA or PO1) showed no autofluorescence at both R- and H-wound sites (Supplementary Fig. S3). All together, these results point out that the fast-developing wound-induced ROS burst, and more specifically H_2_O_2_, could act as an upstream key mediator in both initiating and rescuing regeneration.

### Hydrogen peroxide treatment rescues regeneration in dormant MEK-inhibited fragments

**Figure 2:**
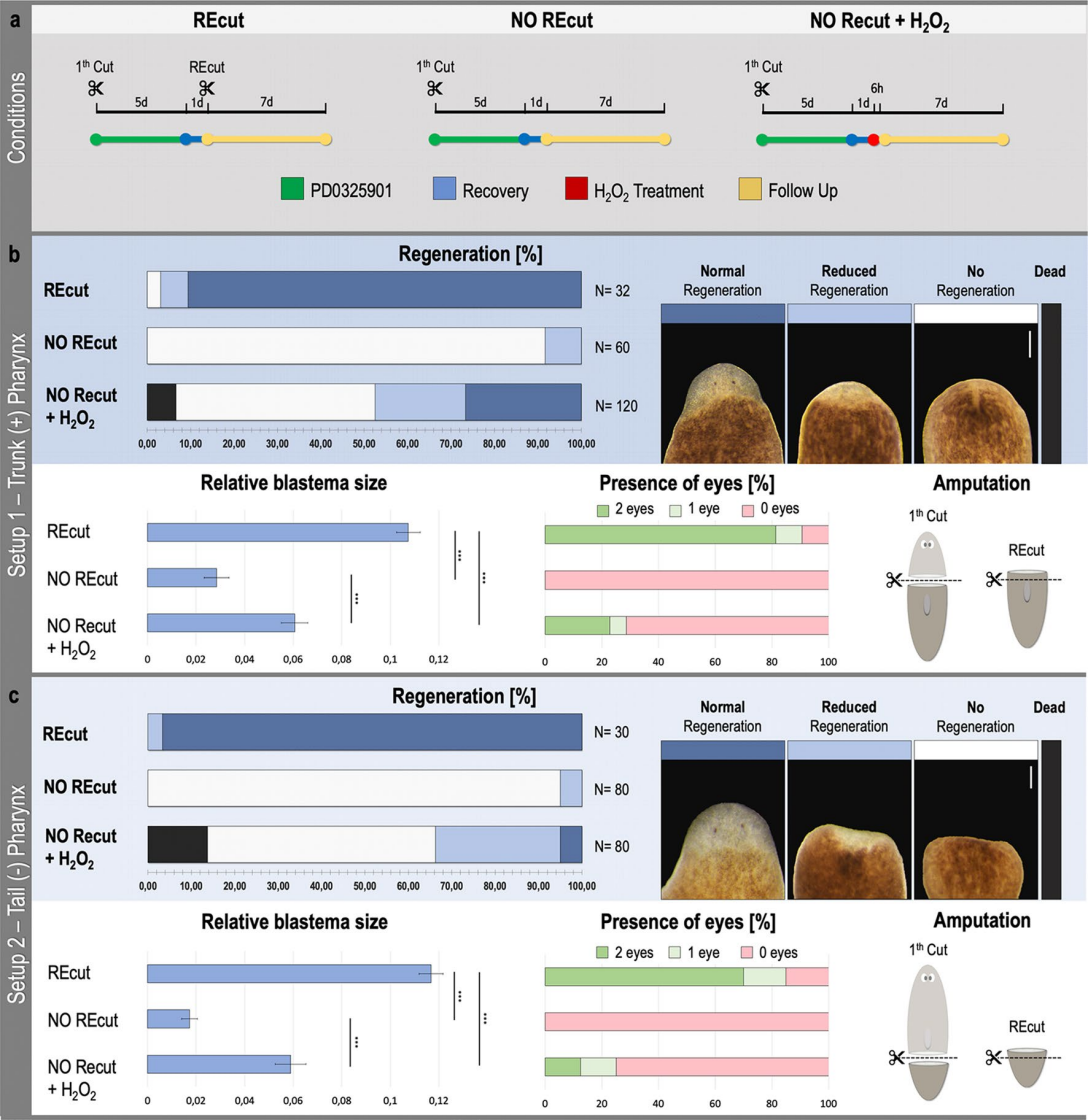
H_2_O_2_ treatment rescues regeneration in MEK-inhibited dormant fragments. (**a**) For each condition indicated above, the experimental setup is displayed. Color code as follows; green: MEK inhibition by PD0325901 (10 μM,5 days), blue: recovery in fresh medium after several washes (1 day), red: treatment with H_2_O_2_ (1.5–2.25 mM, 6 h), yellow: follow up in fresh medium after several washes (7 days). (**b**) Dormant MEK-inhibited trunk fragments with pharynx and (**c**) tail fragments without pharynx were “REcut” or treated with exogenous H_2_O_2_ (“NO REcut + H_2_O_2_”). “NO REcut” controls were neither recut or treated with H_2_O_2_ and were only MEK-inhibited. (**b/c**, upper panel) Graph displaying the percentage of different regenerative outcomes. Sample numbers are indicated in each graph and represent 4 independent experiments. The legend associated with these graphs: no regeneration (white), reduced regeneration (light blue), normal regeneration (dark blue) and dead (black). “Normal regeneration” is considered as full regeneration including both eyes, and if needed pharynx. In the case of “reduced regeneration”, a clear blastema was visible, however, no or one eye(s) and/or no pharynx were differentiated at this time point. “No regeneration” refers to the absence of a blastema and a total block of regeneration. Scale bars 100 µm. (**b/c**, lower left panel) Relative blastema sizes in each condition. Statistical significance is indicated by: *p < 0.05; **p < 0.01; ***p < 0.001. (**b/c**, lower middle panel) Appearance of the eyes. Left bar (green): two eyes; middle bar (light green): 1 eye; right bar (red): no eyes. These graphs represent results of 3 independent experiments with 24 (REcut), 35 (NO REcut) & 56 (NO REcut + H_2_O_2_) biological replicates for the setup using tails with a pharynx (b) and 20 (REcut), 36 (NO REcut) and 38 (NO REcut + H_2_O_2_) for the setup using tails without a pharynx (c). (**b/c**, lower right panel) The amputation setup; 1st cut applied 1 h after incubation in 10 μM PD0325901 followed by 5 days in the same solution, “REcut” applied after recovery period and only applicable in the “REcut” condition). All measurements were carried out 7 days post re-wounding or H_2_O_2_ treatment.

To functionally confirm a role for ROS as a regeneration initiating signal, we investigated if we could reactivate MEK-inhibited, dormant blastemas by treating them with exogenous ROS. Because of the fact that it is present at the wound site post amputation, its vital role in regeneration and the supporting evidence for its activating role in several molecular pathways, we used the non-radical ROS, H_2_O_2_, as exogenous ROS-source and possible regeneration initiating signal (Fig. 2). When inflicting a new R-wound (Fig. 2b: REcut) to MEK-inhibited trunk fragments (with pharynx), 96.88% (n = 32) of the fragments regenerated. A small fraction of these animals (6.25%) started regenerating but experienced a reduced eye development at the moment of comparison. In the absence of a new R-wound (Fig. 2b: NO REcut), 91.67% (n = 60) of the MEK-inhibited trunks failed to regenerate, while the remaining part regenerated a smaller blastema. In tail fragments (without a pharynx), 100% (n = 30) restarted regeneration after inflicting an R-wound, of which 96.67% regenerated normally while the remaining part failed to regenerate both eyes and/or pharynx (Fig. 2c: REcut). Without inflicting a new R-wound, 95% (n = 80) of the fragments did not regenerate and in 5% regeneration was impaired (Fig. 2c: NO Recut). In all “NO Recut” cases of both setups, the absence of regeneration remained for at least 21 days. Initial dose–response experiments indicated that a 6-h treatment with 1.5 to 2.25 mM H_2_O_2_ was enough to fully rescue 26.76% (n = 120) of the MEK-inhibited trunk fragments (with pharynx), and 5% (n = 80) of MEK-inhibited tail fragments (without pharynx) in the absence of re-amputation. An additional 20.83% of the trunk fragments were partially rescued, showing an impaired regeneration. In case of the tail fragments this partial rescue reached 28.75% (Fig. 2b/c: NO REcut + H_2_O_2_). In both setups, a small number of animals died, being 6.58% and 13.75% for respectively trunk and tail fragments. We quantified the rescue in regeneration after H_2_O_2_ treatment by measuring the blastema relative to the whole fragment. H_2_O_2_ treatment after MEK-inhibition led to significantly bigger blastema sizes compared to MEK-inhibited regenerating fragments without such treatment. Furthermore, recutting dormant fragments led to significantly bigger blastemas compared to the H_2_O_2_-treated and the MEK-inhibited fragments without further treatment (Fig. 2b/c). The same trends were observed for the differentiation of new eyes (Fig. 2b/c).

In order to exclude that the observed rescue was induced by tissue wounding as a result of the H_2_O_2_ treatment, a ROS staining was performed on dormant fragments after 3 and 6 h of exposure to H_2_O_2_. The epidermis maintained its integrity, at least at the morphological level. Neither epidermal wounds, nor ROS signals were observed in these H_2_O_2_-treated planarians (Supplementary Fig. S4). Taken together, these results show the probable role of H_2_O_2_ as an upstream regeneration-initiation signal and its ability in rescuing dormant MEK-inhibited fragments.

### Light therapy rescues regeneration in dormant MEK-inhibited fragments

**Figure 3:**
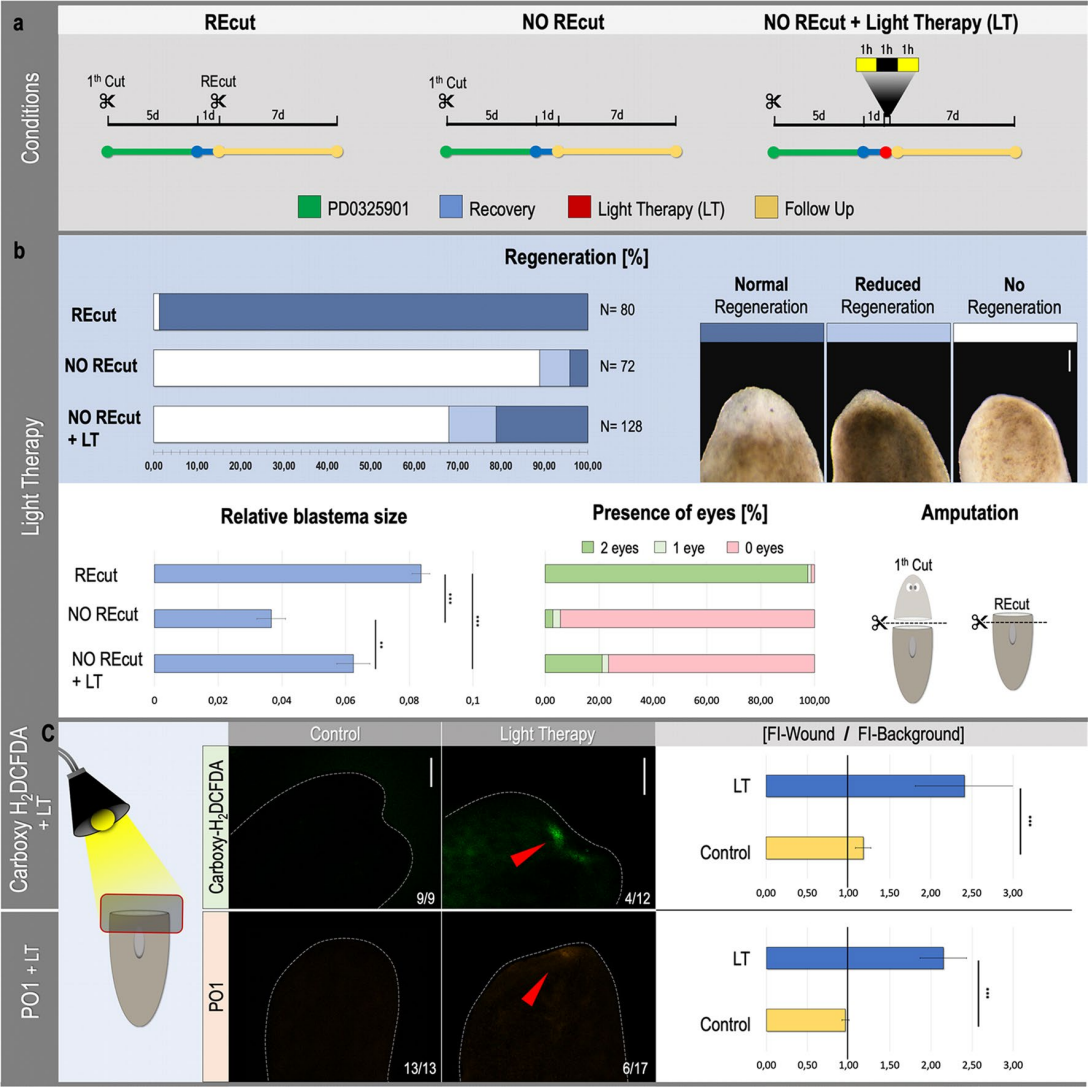
Light therapy rescues regeneration in MEK-inhibited dormant fragments. (**a**) For each condition the experimental setup is displayed. Color code as follows; green: MEK inhibition (PD0325901, 10 μM, 5 days), blue: recovery in medium (1 day), red: light therapy (VIS light, 3 h; 1 h light-1 h dark-1 h light), yellow: follow up in medium (7 days). (**b**) Dormant MEK-inhibited trunk fragments with pharynx were “REcut” or treated with light therapy “LT”. “NO REcut” controls were neither recut or treated with light therapy and were only MEK-inhibited. (**b**, upper panel) Graph displaying the percentage of different regenerative outcomes. Sample numbers are indicated in each graph and represent 5 independent experiments. At the upper right, the legend associated with the graph: no regeneration (white), reduced regeneration (light blue), normal regeneration (dark blue). “Normal Regeneration” is considered as full regeneration including both eyes. In the case of “Reduced Regeneration”, a clear blastema was visible, however, no or one eye(s) were differentiated at this time point. “No Regeneration” refers to the absence of a blastema and a total block of regeneration. Scale bars 100 µm. (**b**, lower left panel) Relative blastema sizes per condition. Statistical significance is indicated by: *p < 0.05; **p < 0.01; ***p < 0.001. (**b**, lower middle panel) Appearance of the eyes. Left bar (green): two eyes; middle bar (light green): 1 eye; right bar (red): no eyes. This graph represents the same samples as used to measure the blastemas. (**b**, lower right panel). The amputation setup; 1^st^ cut applied 1 h after incubation in 10 μM PD0325901 followed by 5 days in the same solution, 2^nd^ cut applied after the recovery period and only applicable in the “REcut” condition. All measurements were carried out 7 days post rewounding or light therapy. (**c**, left panels) in vivo ROS stain (Carboxy-H_2_DCFDA: general ROS, PO1: hydrogen peroxide) after conducting light therapy on MEK-inhibited tails. Controls were stained after the recovery period, without light therapy while treated samples were directly stained after light therapy. A clear ROS production (general ROS + hydrogen peroxide) was visualized after light therapy, at the original R-wound applied before MEK-inhibition (red arrowhead). Sample numbers are indicated in the images. All pictures were taken under the same light intensity settings. The dotted, white lines indicate the border of the wound site. Scale bars 100 µm. (**c**, right panels) Relative fluorescence intensity at the original R-wound region. Upper graph indicates relative fluorescence intensity representing general ROS production. Lower graph indicates relative fluorescence intensity representing hydrogen peroxide production. Statistical significance is indicated by: *p < 0.05; **p < 0.01; ***p < 0.001.

Light or photomodulation therapy (LT) is broadly applied in certain pathologies to stimulate wound healing and stem cell activity. Several publications associated this stem cell stimulation with a therapy-induced ROS production, activating underlying signaling pathways^32–37^. We investigated the possibility of rescuing dormant MEK-inhibited fragments by an indirect way of ROS production, in this case light therapy. As already confirmed in Fig. 2b, recutting MEK-inhibited trunk fragments led to a full regeneration in the majority of the fragments (98.75%; n = 80), while in the absence of a new R-wound the fragments remained dormant (88.89%; n = 72) (Fig. 3b: REcut and NO REcut). On the other hand, treating dormant, MEK-inhibited fragments with light therapy was sufficient to rescue regeneration in 32.09% of them from which 21.09% fully regenerated (n = 128). The other 67.97% was not able to regenerate (Fig. 3b: NO Recut + LT). The extent of this rescue was quantified by measuring blastema size with respect to the size of the whole fragment. Remarkably, light therapy after MEK-inhibition led to significantly bigger blastemas compared with blastemas from MEK-inhibited regenerating fragments without any treatment. Re-cutting dormant fragments led to significantly bigger blastemas compared with the light treated ones as well as the MEK-inhibited fragments without any treatment. The same trends were observed for the differentiation of the eyes (Fig. 3b).

To test the assumption of ROS as a possible underlying mechanism in rescuing regeneration in MEK-inhibited fragments, an in vivo general ROS staining was carried out after the light therapy. A total of 30% (n = 12) of the fragments treated with light therapy presented a clear ROS production at the original, dormant, R-wound site while a slight ROS production was visualized in the rest of the body (Fig. 3c, left panels, red arrowhead). Strongly corresponding results were obtained after conducting the more specific in vivo H_2_O_2_ stain. In roughly 35% (n = 17) a clear H_2_O_2_ production was visible at the original R-wound after applying light therapy (Fig. 3c, left panels, red arrowhead). The percentage of fragments displaying a clear ROS/H_2_O_2_ production is in accordance with the percentage of animals in which regeneration was rescued after light therapy (Fig. 3b: LT). To confirm that the ROS production at the original R-wound site was induced by light therapy, a ROS staining (general ROS and H_2_O_2_) was performed on dormant fragments without light treatment. In this condition, no ROS or H_2_O_2_ production was observed in any planarian (general ROS: n = 9, H_2_O_2_: n = 13) (Fig. 3c, control). Quantification of the fluorescent signal showed a statistically significant increase in both ROS and H_2_O_2_ production induced by light therapy (Fig. 3c, right panels). Additionally, temperature of the medium was measured before and after applying the light therapy. This was performed in order to exclude that the rescue was not induced by oxidative stress caused by the heating up of the medium in which the MEK-inhibited fragments were put during the light therapy. Before and after the light therapy the medium was exactly at the same temperature of 18 °C. Supplementary Figure S5a displays a possible mechanism by which ROS can be produced after applying light therapy. Stubenhaus and colleagues proposed this model in which porphyrin, present in pigment cells, produces ROS after photomodulation, which in turn can activate intracellular signaling cascades or diffuse out of the cell and trigger surrounding cells instead^36,37^. Directly after applying light therapy, worms started to depigment. The depigmentation increased over time. This can be observed in supplementary Fig. S5b when comparing the pigmentation level on day 4 and day 7 post light therapy (DPLT). With these results we point out the possibility of photomodulation-induced rescue of MEK-inhibition and subsequent rescue of regeneration. We hypothesize ROS production after applying light therapy, as underlying mediator, functioning to (re)stimulate stem cell activity.

### Inhibition of ROS production blocks ERK activation at the wound site

**Figure 4:**
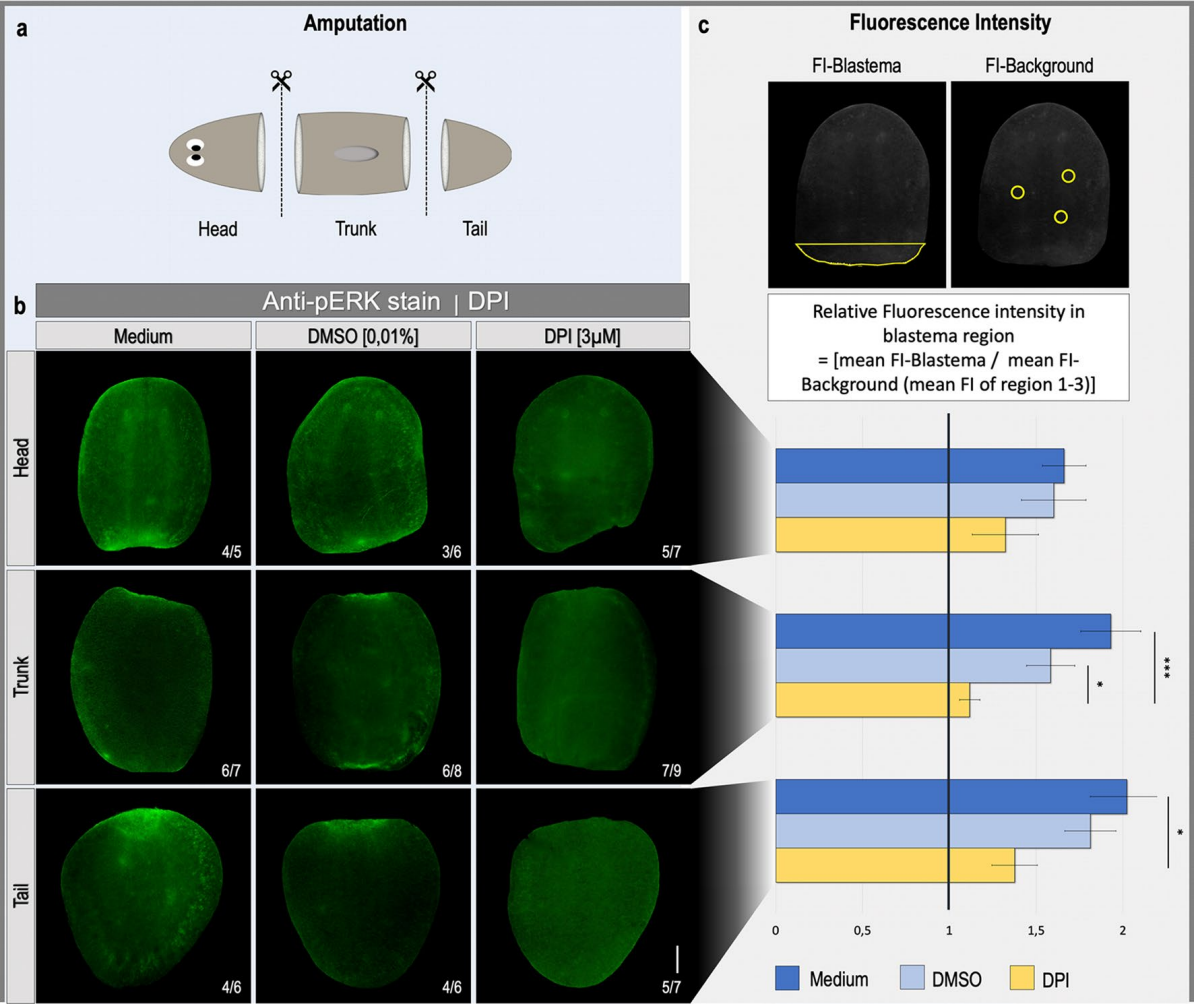
pERK activation decreases after the inhibition of ROS production (**a**) The amputation setup is displayed in the light blue box, indicating the amputation sites. (**b**) Immunostaining with an anti-pERK antibody on 1-day regenerating fragments. Animals kept in culture medium or 0.01% (v/v) DMSO were used as control animals, while in the treatment group, ROS production was inhibited with DPI (3 μM) administered in the culture medium during regeneration. All pictures were taken under the same light intensity settings. Sample numbers are indicated in the images. Scale bars 100 µm. (**c**) Relative fluorescence intensity of the blastema region. Setup of the measurement is indicated in the upper panel while the mean relative fluorescence intensity is displayed in the graph below. Color code as follows; dark blue: control in culture medium, light blue: control in 0.01% DMSO, yellow: 3 μM DPI. Statistical significance is indicated by: *p < 0.05; **p < 0.01; ***p < 0.001.

To identify whether in normal conditions the amputation-induced ROS production is required for the activation of the ERK signaling pathway, we performed an immunostaining with anti-pERK antibody after ROS inhibition by DPI^26,38^. When animals were kept in regular medium or DMSO, a clear activation of pERK was observed at the wound site of head- (control: 4/5, DMSO: 3/6), trunk- (control: 6/7, DMSO: 6/8) as well as tail pieces (control: 4/6, DMSO: 4/6). This pERK-signal was strongly reduced when fragments were treated with DPI (heads: 5/7, trunks: 7/9, tails: 5/7) (Fig. 4b). After quantification of the signal attributed to activated ERK, statistical significance was obtained between both control conditions (medium and DMSO) and ROS inhibited trunks as well as between control- (medium) and ROS-inhibited tails (Fig. 4c). Despite no other significant difference, a strong trend was visible between both control conditions (medium and DMSO) and ROS-inhibited fragments of either heads, trunks or tails. Taken together, these data suggest that an amputation-induced ROS production at the wound site is required for the phosphorylation and proper activation of ERK.

### *Smed-egfr-3* silencing impairs ROS production at the wound site

**Figure 5:**
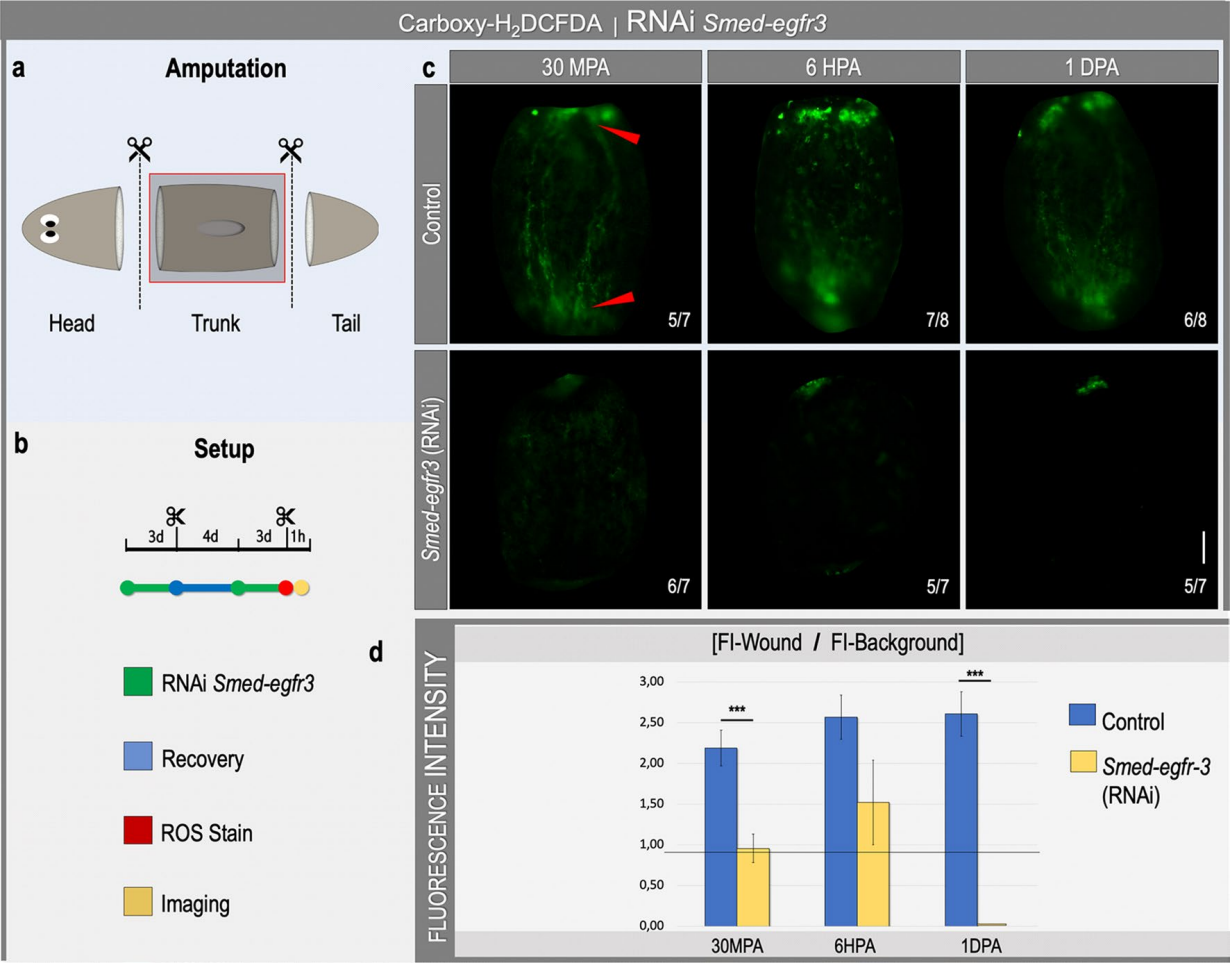
ROS production is regulated by *Smed-egfr-3* during early regeneration. (**a**) The amputation setup is displayed in the grey box, indicating the amputation sites. The red square corresponds to the images shown in (c). (**b**) The experimental setup is displayed in the light grey panel. Color code as follows; green: *Smed-egfr3* RNAi (2 rounds of 3 consecutive days), blue: recovery in fresh medium (4 days), red: general ROS stain using carboxy-H_2_DCFDA, yellow: imaging procedure. (**c**) In vivo visualization of amputation-induced ROS levels 30 min-, 6 h- and 1 day post amputation (MPA, HPA, DPA) in controls and *Smed-egfr-3* RNAi fragments. All pictures were taken under the same light intensity settings. Sample numbers are indicated in the images. Scale bar 100 µm. (**d**) Relative fluorescence intensity of the wound region. Color code as follows; dark blue: control, yellow: *Smed-egfr-3* (RNAi). Statistical significance is indicated by: *p < 0.05; **p < 0.01; ***p < 0.001.

In literature, many examples exist of the activation of the epidermal growth factor receptor (EGFR) signaling by ROS and the role of EGFR in mediating MAPK signaling including ERK activation^26–31,39,40^. To further characterize the relationship between ROS and the EGFR/MAPK signaling during planarian regeneration, an in vivo ROS visualization was performed in controls and animals subjected to RNAi-mediated silencing of *Smed-egfr-3* (Fig. 5). Whereas the presence of the amputation-induced ROS burst was clear in the control animals (30MPA: 5/7, 6HPA: 7/8, 1DPA: 6/8), trunk fragments subjected to *Smed-egfr-3* knockdown showed a clearly diminished ROS production at all time points (30MPA: 6/7, 6HPA: 5/7, 1DPA: 5/7) (Fig. 5c). Quantification of the fluorescence intensity confirmed the statistically significant decrease in ROS production after knocking down S*med-egfr-3* (Fig. 5d). These results indicate that *Smed-egfr-3* might play a pivotal role in regulating the amputation-induced ROS production, possibly through the existence of a feedback mechanism. Furthermore, it is suggested that the activation of pERK by ROS could be mediated by the EGFR pathway during regeneration.

In addition, we explored the possibility of aquaporins functioning as H_2_O_2_ transporter, playing an essential role in redox-related activation of the Smed-egfr-3/MAPK pathway. Seven planarian aquaporins were identified and co-expression with ERK, EGFR-3 and some of these aquaporins were observed in neoblasts, neurons and epidermal cells after in silico analysis. However, no regeneration defects were observed after knocking down the individual aquaporins (Supplementary Fig. S6 and S7).

### Knocking down *Smed-egfr-3* diminishes pERK activation in regenerating animals

**Figure 6:**
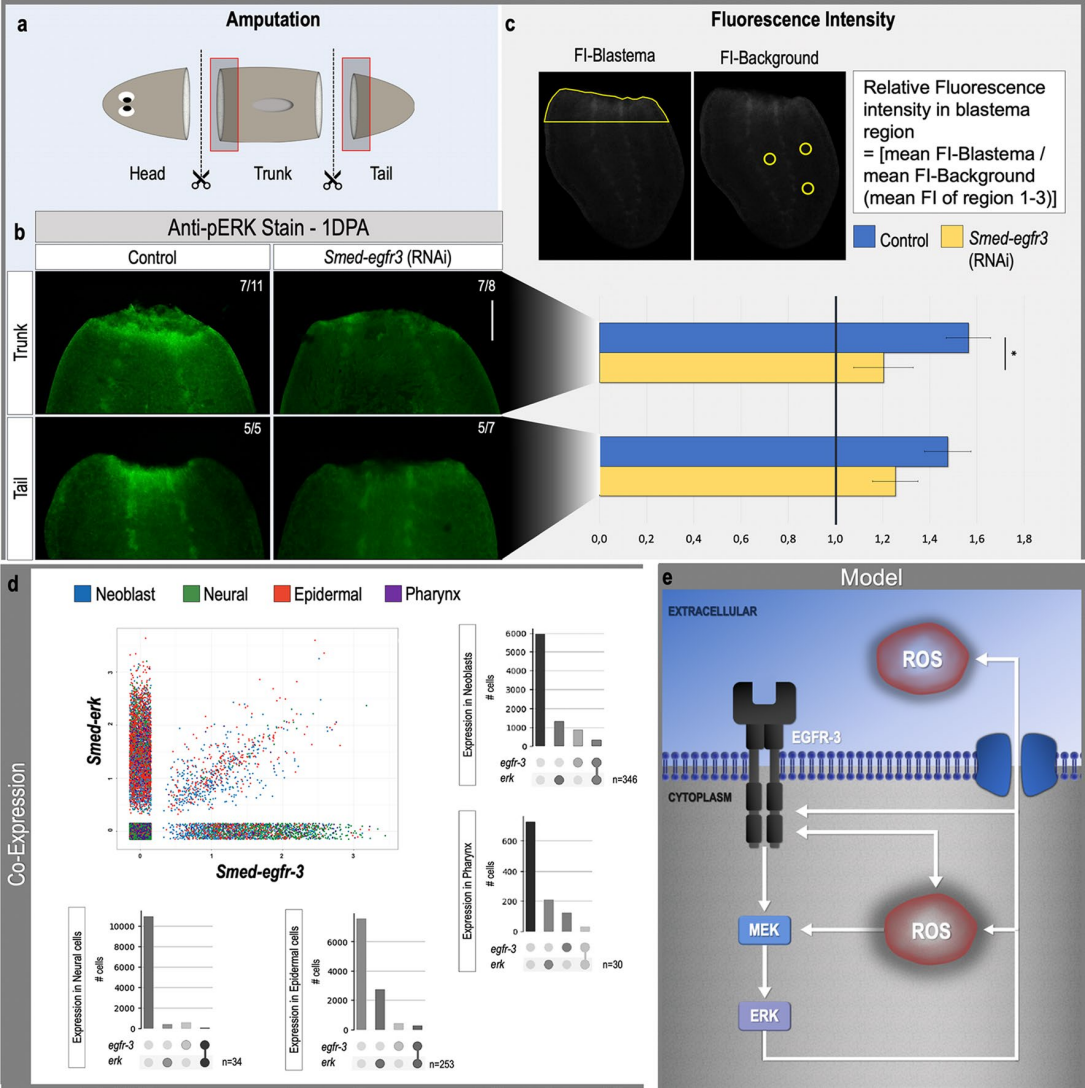
*Smed-egfr-3* is required for pERK activation. (**a**) The amputation setup is displayed in the light blue box, indicating the amputation sites. Red squares correspond to the anterior blastemas shown in B (**b**) Immunostaining with an anti-pERK antibody in controls and after *Smed-egfr-3* RNAi at 1 day post-amputation. All pictures were taken under the same light intensity settings. Sample numbers are indicated in the images. Scale bars 100 µm. (**c**) Relative fluorescence intensity of the blastema region. Setup of the measurement is indicated in the upper panel while the mean relative fluorescence intensity is displayed in the graph below. Color code as follows; dark blue: control, yellow: *Smed-egfr3* RNAi. Statistical significance is indicated by: *p < 0.05; **p < 0.01; ***p < 0.001. (**d**) Expression and co-expression of *Smed-erk* and *Smed-egfr-3* based on single cell sequencing available^41,42^. For each of the lineages analyzed in each graph the first column represents the number of cells of that particular lineage in which neither *Smed-egfr-3* nor *Smed-erk* are expressed. The second column indicates the number of cells expressing *Smed-erk*. The third column indicates the number of cells expressing *Smed-egfr-3*. Finally, the fourth column indicates the number of cells in which *Smed-egfr-3* and *Smed-erk* are co-expressed. (**e**) A possible regulatory pathway implicating ROS, Smed-EGFR-3, MEK and ERK is displayed based on literature and the results presented here.

In planarians, silencing *Smed-egfr*-3 results in impaired regeneration with impaired stem cell differentiation^30^. In addition, previous results suggested a relationship between *Smed-egfr-3* and ERK activation^26^. To further investigate the functional relationship between *Smed-egfr-3* and pERK, we carried out an immunostaining with the anti-pERK antibody in controls and RNAi-mediated *Smed-egfr-3* knockdown animals (Fig. 6a). A clear pERK activation was observed in all control fragments (Fig. 6b, trunk pieces: 7/11, tail pieces: 5/5). In contrast, a strong reduction of the anti-pERK signal at the wound site was detected in *Smed-egfr-3* silenced fragments (Fig. 6b, trunk pieces: 7/8, tail pieces: 5/7). After quantification of the fluorescent signal indicating activated ERK, statistical significance was determined between controls and *Smed-egfr-3* knockdown trunk pieces. Despite no other significant difference, the same trend was observed between controls and *Smed-egfr-3*-RNAi tail pieces (Fig. 6c).

To check whether *Smed-egfr-3* and *Smed-erk* are indeed expressed in the same cell types, we analyzed the expression of these two genes at the single cell level at the online resource at digiworm.wi.mit.edu^41^ and used the tools in PlanExp^42^ to visualize their co-expression. *Smed-egfr-3* and *Smed-erk* are co-expressed in a variety of cell types including neoblasts, neurons, epidermis and pharyngeal cells (Fig. 6d). Overall these results suggest that *Smed-egfr-3* could work as an upstream factor for the activation of ERK. In Fig. 6e we summarized the interactions between ROS and the *Smed-egfr-3*/MAPK pathway, based on all results presented in this paper and supplemented with literature.

## Discussion

The complex process of animal regeneration is highly organized and regulated by a tightly controlled network of signaling pathways, of which we have only discovered the tip of the iceberg. Regeneration research has mainly focused on deciphering the molecular mechanisms regulating cell fate, polarity re-establishment, tissue differentiation and organ positioning; however, the question on which initial signals trigger regeneration remains poorly understood. In recent years, several studies have reported the indispensability of reactive oxygen species (ROS) during regeneration. Amputation-induced ROS production was observed in early tail regeneration of *Xenopus* tadpoles and in fin regeneration of adult zebrafish^20,21^. Similar observations were reported during the regeneration process of the wing imaginal disc in *Drosophila* and during regeneration of the planarian *Schmidtea mediterranea*^17,22^. In all of the above-mentioned cases, ROS production was clearly linked to the capacity to regenerate, e.g. diminished ROS production during regeneration-initiation led to regenerative impairments (Supplementary Fig. S1)^14,17,20–22^.

In a previous study we demonstrated the use of two different ROS-inhibiting compounds in *S. mediterranea*, i.e. DPI and APO^17^. Both drugs are widely used in in vitro models as well as in vivo regeneration models. In *S. mediterranea,* DPI-induced effects were more severe in comparison with APO-related defects, which could be explained by their substrate specificity. While APO specifically blocks NOX-like enzymes, DPI targets both extracellular membrane-associated as well as mitochondrial flavoproteins causing a stronger decrease in ROS production and consequently inducing more severe regeneration defects^43–45^. Therefore, we used DPI to further analyze the functional role of ROS during planarian regeneration. Off-target effects of DPI were taken into account as also other approaches were used to interfere with the redox state, such as light therapy. In addition to the ROS burst observed at the wound site after inflicting a regenerative(R)-wound^17^, our current data also show ROS production after inflicting a healing(H)-wound (Fig. 1a). In both cases, the ROS burst was shown via a general oxidative stress indicator (Carboxy-H_2_DCFDA), and a more specific one (Peroxy Orange 1) to show that H_2_O_2_ is amongst the reactive oxygen species produced (Fig. 1). PO1 is new for planarian research but is extensively used in in vitro as well as in vivo models and is, just as carboxy-H_2_DCFDA, specific for the detection of intracellular species^46–48^. False positives cannot be completely excluded after the use of in vivo ROS stainings, but both DPI and APO strongly decreased the ROS signal and induced similar results in the exposed planarians. Owlarn and colleagues have described that both R-wounds and H-wounds trigger a common initial molecular response mediated by ERK signaling. Inhibition of the ERK upstream activator, MAPK/ERK kinase (MEK), completely blocks regeneration after an R-wound^14^. The body fragments remain “dormant” until a new R- or H-wound is made, which somehow re-activates the regeneration program. The fact that inflicting a new H-wound elsewhere is capable of rescuing regeneration at the original dormant R-wound suggests that, in planarians, any kind of wounding (whether or not it results in tissue loss) triggers an early response through ERK activation^14^. In the current study, we observed a fast production of ROS, and H_2_O_2_ in specific, in these MEK-inhibited dormant fragments after inflicting a new H- or R-wound (Fig. 1b). Surprisingly, H-wounding in the flank not only induced ROS production at the applied H-wound site but also at the original, dormant R-wound site (Fig. 1b). Together with the results of (1) a significant rescue of regeneration at the dormant, MEK-inhibited R-wound site when applying an H-wound elsewhere and (2) a blocked rescue of regeneration when combining the re-wounding of MEK-inhibited tails with DPI treatment (Supplementary Fig. S2), our data indicate a possible involvement of ROS in the activation of the MAPK/ERK pathway.

This is supported by literature, as in many of the addressed cellular events modulated by ROS, MAPKs are predominantly mentioned as a required intermediate link^22–25,31,49–51^. The catabolic cysteine residues, present on cytokine receptors and growth factors upstream of the MAPK/ERK pathway as well as on more downstream MAPK signaling molecules like MAP3Ks, are generally accepted as critical targets of oxidative stress. Several studies demonstrate this link by blocking MAPK signaling after interfering with ROS production or by activating MAPKs after treatment with exogenous H_2_O_2_^23–25,52–54^. Taking into account that, in planarians, ROS are necessary for regeneration and are induced directly after H- and R-wounding, and ERK activation is required for regeneration in the same timeframe and location as we observe with ROS production, we further searched to determine the functional link between ROS and ERK activation. We demonstrated that after adding exogenous H_2_O_2_, MEK-inhibited dormant tails can be rescued without the need to inflict a new wound (Fig. 2). It is important to point out that the H_2_O_2_ treatment did not induce visible epidermal wounding, indicating that the rescue was not attributed to the re-wounding of dormant tails (Supplementary Fig. S4). We hypothesize that because of its relatively long half-life and good membrane permeability, the non-radical ROS, H_2_O_2_, functions as a secondary messenger and triggers the activation of important regeneration-related downstream signaling processes such as ERK activation. We were not able to show a role for aquaporins in this H_2_O_2_ signaling at the initial stage of planarian regeneration. Seven planarian aquaporin genes were identified, but RNAi of the individual aquaporins did not result in any defect in regeneration and CNS formation indicating that there could be some level of redundancy among them (Supplementary Fig. S6). Unfortunately, there is no current way to knockdown all aquaporins simultaneously. In silico analysis did show co-expression of ERK, EGFR-3 and some of the aquaporins in neoblasts, neurons and epidermal cells (Supplementary Fig. S7). Also, H_2_O_2_ is able to cross cell membranes on its own or via hydrophobic pores induced by configuration changes of oxidized lipids initiated via intra- and intermolecular processes^19,55–57^.

The strongly corresponding regeneration impairments (Supplementary Figure S1) when interfering with the regeneration-induced ROS burst, or with the ERK activation^17,38^, again show an interaction between both events. Previously, defects in neoblast differentiation and a significant decrease of the wound-induced expression of secreted frizzled related protein 1 (sfrp1) was shown after the inhibition of ROS production and blocking ERK activation^17,38^. It is believed that the expression dynamics of sfrp1 are regulated by the activity levels of ERK^38,58^. Together with the strong reduction of ERK activation at the wound site of regenerating animals after inhibition of ROS production (Fig. 4), we hypothesize an upstream function of ROS relative to ERK and its ability to activate the MAPK/ERK signaling pathway during regeneration initiation. However, we have to take into account that MAPK/ERK are activated as a response to many different signals and it cannot be excluded that ROS do not act alone but as a part of a more complex initiation signaling system^59,60^.

To validate H_2_O_2_ as potential regeneration-initiator, reactivating MAPK/ERK signaling, and to exclude the possibility of regeneration rescue due to secondary wounding, we investigated a rather indirect way of rescuing regeneration. Instead of direct exposure to exogenous H_2_O_2_, we explored the possibility of using photomodulation therapy, in this study referred to as light therapy, which is a widely accepted tool to treat pathological tissues such as different wounds to control inflammatory processes, and also to promote tissue healing^32^. Photomodulation therapy induces photochemical changes and stimulates stem cell activity by increasing proliferation, migration and differentiation^32,33,61^. The commonly proposed mechanism behind it is that the absorbed energy of the light therapy induces an increased cytochrome c oxidase enzyme activity, electron transportation, oxidative respiration, membrane potential and ATP production, leading to an increased ROS production, cytokines and expression of growth factors. In turn this can lead to the initiation of several signaling cascades, promoting cellular proliferation, migration and differentiation^32–34,62^. In many of these studies, it is believed that ROS-stimulated MAPK–ERK signaling is one of the important, downstream activated signaling pathways^35^. For example, the application of light therapy to human keratinocytes, using visible light, leads to ROS-induced phosphorylation and subsequently activation of EGFR-ERK signaling pathway^63^. However, as indicated by Stubenhaus et al.^36^, exposing *S. mediterranea* to intense light can lead to ROS production by another mechanism (Supplementary Fig. S5a). Intense light exposure causes porphyrin, produced in planarian pigment cells, to generate ROS. ROS then drives the oxidation of unoporphyrinogen I, leading to more ROS production, generating a positive feedback loop and eventually causing pigment cell loss^36,37^. We hypothesized that these ROS can subsequently activate the aforementioned MAPK–ERK pathway in exactly the same way. Similar analytical results of porphyrin-induced photogeneration of H_2_O_2_ are observed in several in vitro studies^64,65^. We achieved a remarkable percentage of regeneration rescue, similar to the obtained rescue when treated with exogenous H_2_O_2_ (Fig. 3). After light therapy, MEK-inhibited tails strongly depigmented, illustrating the presence and production of ROS as described earlier (Supplementary Fig. S5)^34–37^. This assumption is confirmed by the slight overall presence of ROS, and H_2_O_2_ specifically, with a distinguishable and significantly stronger signal of both general ROS and H_2_O_2_ at the original dormant R-wound site after light therapy (Fig. 3c). Overall, these results demonstrate light therapy as a useful tool in re-initiating regeneration after inhibiting MAPK–ERK pathway and confirm the potential of photomodulation as application in regenerative medicine. The exact mechanism, however, remains to be elucidated. Although ROS and H_2_O_2_ are produced directly after inducing regeneration and before the first apoptotic peak, we cannot exclude that both exogenous H_2_O_2_ treatment or intense visible light can induce regeneration via apoptosis-induced proliferation^66,67^.

In many organisms, the epidermal growth factor receptors (EGFR), one of families of the receptor tyrosine kinases (RTK), are known to regulate several biological processes by activating key downstream pathways including the MAPK pathway^28,29,33,39^. In planarians, *Smed-egfr-*3 is required for proper regeneration as well as for ERK activation (Fig. 6b)^26^. Recent literature. suggested that RTK-associated activation mechanisms are under strict redox control. Activation of EGFR signaling by ROS can occur in several ways^30^. On the one hand, intracellular ROS can facilitate the phosphorylation of EGFR and induce a subsequent cascade of phosphorylation of downstream elements. According to Peus and colleagues, H_2_O_2_ specifically acts as a critical mediator in this case^68^. The absence of ERK activation after inhibition of ROS production (Fig. 4), and the rescue of regeneration in dormant tails by either H_2_O_2_ or light therapy in our results support this hypothesis (Figs. 2, 3). Furthermore, silencing Smed-egfr-3 results in significantly decreased amputation-induced ROS production, suggesting the dependency of ROS production on the EGFR signaling (Fig. 5). This could be explained by the fact that ligand-dependent dimerization of EGFR induces ROS production for its autophosphorylation and consequent activation, leading to a decreased intracellular ROS production when silenced^30^. However, literature also suggests that H_2_O_2_ acts as a critical mediator in the ligand-independent phosphorylation and activation of EGFR^68^. On the other hand, the regulation of NADPH-oxidases (Nox) expression can be controlled by ERK-activation and the subsequently activated transcription factors. Knocking down *Smed-egfr-3*, which led to a decreased ERK activation, could therefore alter Nox expression, explaining the impaired ROS production^30,69^. However, the involvement of Nox genes in planarians still has to be elucidated as no homologues have been identified yet in the current genomic and transcriptomic databases. In addition, future research is necessary to further explore and describe these feedback mechanisms more in detail.

On the other hand, as MEK is located downstream of EGFR, we would expect a decreased ROS/H_2_O_2_ production at the wound site after applying wounds in MEK-inhibited tails. However, no significant decrease in ROS/H_2_O_2_ signal was observed (Fig. 1d). This could be explained by (1) the presence of MEK-independent ROS producing mechanisms, as well as (2) the possible occurrence of redundancy. Altogether we propose a signaling model of interactions between ROS and the EGFR–MAPK–ERK pathway that is addressed during the earliest phase of planarian regeneration. The regulatory pathway suggested here is based on relevant literature on planarians and other systems and is complemented with results presented in this paper (Fig. 6e).

In summary, our results suggest that: (1) ROS and/or H_2_O_2_ have the potential to rescue regeneration in MEK-inhibited dormant tails, (2) ROS and/or H_2_O_2_ are required for ERK activation at early regeneration stages, (3) the EGFR pathway can mediate ROS production with ERK activation during planarian regeneration. We provide the first evidence of amputation- and wound-induced ROS/H_2_O_2_ production in upstream relationship with the EGFR–MAPK–ERK signaling pathway during planarian regeneration in which ROS are not only identified as most upstream trigger for regeneration-initiation, but could possibly perform its functions also more downstream. In the future, there is a need for next-generation-specific ROS probes that will allow for a better report on both location and nature of inter- and intracellular production of specific forms of ROS. This will help in exploring the putative role of Noxes, the involvement of aquaporins and the ROS-induced MAPK/ERK activation, during planarian regeneration. It is necessary to further investigate the relationships of ROS with other signaling cascades, which might help in the understanding of how ROS signaling could be manipulated in order to improve regeneration in other models and humans.

## Methods

### Planarian cultivation

An asexual strain of the freshwater planarian species *S. mediterranea* was kept in Milli-Q water containing 1.2 mM NaHCO_3_, 1.6 mM NaCl, 1.0 mM CaCl_2_, 1.0 mM MgSO_4_, 0.1 mM MgCl_2_ and 0.1 mM KCl. Planarians were continuously maintained in the dark at a temperature of 20 °C. Once a week they were fed with veal liver. Animals used in experiments were starved for at least 7 days before the procedure.

### Inhibition of reactive oxygen species (ROS) production

The nonspecific flavoprotein inhibitor, Diphenyleneiodonium chloride (DPI, Sigma Aldrich, D2926), was used in order to block ROS production by interfering with several electron transporters. Animals were exposed to 3 μM DPI for 5 h prior to in vivo ROS staining or inflicting a second cut in MEK-inhibited tails, and 1 h prior to amputation when followed by pERK immunohistochemistry. In both cases, animals were continuously exposed to DPI during the regeneration period. Because of its hydrophobic character, DPI was prepared in 0.01% (v/v) dimethylsulfoxide (DMSO, Sigma Aldrich, 471267). In all experiments using DPI, a DMSO-exposed control group was added to take into account the possible effects of DMSO since relatively high concentrations can have neurotoxic effects and influence cell proliferation in *S. mediterranea*^70^.

### MEK inhibition

The chemical compound PD0325901 (Calbiochem) was used to reversibly inhibit MEK activity and subsequently prevent the activation of ERK. As a consequence, we obtained dormant planarian fragments as described by Owlarn et al.^14^. PD0325901 was dissolved in DMSO and used in a concentration of 25 μM. Planarians were exposed to PD0325901 for 1 h prior and up to 5 to 7 days post amputation. The exposure solution was replaced daily. After treatment with PD0325901, animals were gently washed and placed in fresh medium.

### H_2_O_2_ treatment

Dormant, MEK-inhibited fragments were exposed to H_2_O_2_ (Sigma Aldrich, 30% (v/v) in water) with the intention to rescue regeneration. After initial range finding experiments, fragments were exposed to either 1.5 mM (0.005%) or 2.25 mM (0.0075%) H_2_O_2_ (in cultivation medium) for 6 h. Afterwards they were washed for 3 times and kept in fresh medium. All treated samples were handled very carefully in order to prevent wounding.

### Light therapy

MEK-inhibited tail fragments were treated with light therapy in order to investigate the possibility of rescuing regeneration at the original applied R-wound. After initial trail experiments using different wavelengths, the full spectrum of visible light (VIS) was used for the therapy. SCHOTT KL 1500 lcd with a halogen lamp, color temperature of 2950 K and light intensity of 6.500 lx was used to apply light therapy to the MEK-inhibited fragments. Samples were treated with VIS light for two times 1 h each separated by 1 h of recovery in full darkness. Control animals were kept in the dark. Light intensity was measured using the LI-COR quantum/radiometer/photometer (model LI-189), in exactly the same experimental setup as used for the light therapy. All treated samples were handled very carefully in order to prevent wounding.

### RNA interference

Double-stranded RNA (dsRNA) for *Smed-egfr-3* and *Smed-aqp-(1–7)* were synthesized as previously described (*Smed-egfr-3*, forward primer: GTACTGGGCAATGTTGGACCTGGC, reverse primer: TGACGGCCTCATGTGGGGATCATCG; *Smed-aqp-1*, forward primer: GCAGAACTTCTTGGCACCTT, reverse primer: CCCACTATTGGTATCATGGC; *Smed-aqp-2*, forward primer: TTTTGGTTGTCAGTGGTCGC, reverse primer: CGGAAGAGGGAAAACTGACG; *Smed-aqp-3*, forward primer: TTGCCTCAATCGGTCGTTTG, reverse primer: ATGCTCCGAAAACTCCTCCA; *Smed-aqp-4*, forward primer: CGTGGGTCCAATTTCAGG, reverse primer: GGGTACTTTCTATTCGTGAAG; *Smed-aqp-5*, forward primer: GACACATCAATCCAGCCGTC, reverse primer: CGTCATACCGATCAGCCTTT; *Smed-aqp-6*, forward primer: CGTTGAATTCCTAGGAACTTTC, reverse primer: GCCACAATGTTGAGCAATGAC; *Smed-aqp-7*, forward primer: TTCCCTTCACCAACAGCAGG, reverse primer: CCGTGAGCAACGGCAACGGC). Animals were injected in two rounds of 3 consecutive days each with 4 days elapsed in between. On day 4 of the second round, planarians were amputated pre- and post-pharyngeally to induce regeneration. Injections were done using the Nanoject II (Drummond Scientific, Broomall, PA, USA) and consisted of three times 32 nl containing 1 μg/μl dsRNA^71^. Controls were injected with dsRNA of gfp.

### Morphological characterization

The effects of DPI, H_2_O_2_ treatment or light therapy on the ability to restore the regenerative capacity after reversible MEK-inhibition were studied by measuring differences in blastema sizes 7 days post re-cutting (2nd cut) or treatment, compared with MEK-inhibited controls without any treatment. In case of DPI treatment, blastema sizes were measured on day 10 post re-cutting. Blastema areas were quantified using ImageJ (version 1.48v) on digital micrographs acquired with a Nikon DS-Ri2 digital camera mounted on a Nikon SMZ800 stereomicroscope. The blastema area measurements were normalized against the total body area of the worm. These worms were also scored according to their photoreceptor (eye) development. Therefore, the presence of the eyes (2, 1 or 0 eyes) on 7 or 10 days post re-cutting/treatment was recorded.

### Immunohistochemistry

To analyze the relationship between ROS and *Smed-egfr-3* relative to ERK activation in early regeneration, a pERK immunostaining was performed after interfering with the aforesaid. Planarians were fixed at 6 and/or 24 h of regeneration and processed as previously described^26^. Bleached animals were washed with PBSTx (1 × PBS (10 × PBS: 1.37 M NaCl, 27 mM KCl, 100 mM Na2HPO4, 20 mM KH2PO4 in ultrapure H_2_O), 0.3% (v/v) Triton X-100) and incubated for 4 h in 1% blocking solution (1% (w/v) BSA in PBSTx) followed by the primary antibody (anti-pERK) diluted 1/1000 in blocking solution) overnight at 4 °C.

The immunostainings with anti-phospho-histone 3, anti-SYNAPSIN and anti-arrestin VC1 were carried out as described previously^72^. We used anti-phospho-histone3 (PH3, Cell signalling technology) to detect mitotic cells (diluted 1/300); anti-SYNAPSIN used as a pan-neural marker (diluted 1/50, Developmental Studies Hybridoma Bank)^73^; and, anti-VC-1, arrestin (VC-1, a mouse antibody specific for planarian photosensitive cells (1:15,000)^74^. After PBSTx washes and 1 h in blocking solution, they were incubated with the secondary antibody (goat anti-rabbit-POD diluted 1/500 in blocking solution) overnight at 4 °C. After PBSTx washes, samples were incubated for 8 min in TSA Plus Fluorescein solution (1/50 TSA Plus Fluorescein in 1 × Amplification Buffer (Tyramide Signal Amplification Labeling Kit No. 2; Molecular Probes, Thermo Fisher Scientific) in darkness. Samples were mounted after the final PBSTx washes (RT) and analysed with a MZ16F fluorescence stereomicroscope (Leica) equipped with a ProgRes C3 camera (Jenoptik, Jena, Germany).

### Fluorescence intensity measurements of general ROS-, H_2_O_2_- or pERK signal

The relative fluorescence intensities of samples with an in vivo general ROS or H_2_O_2_ staining as well as samples with a pERK immunostaining, were quantified using ImageJ (version 1.48v) on images taken with a MZ16F fluorescence stereomicroscope (Leica) equipped with a ProgRes C3 camera (Jenoptik, Jena, Germany). The mean intensity values were obtained for anterior and/or posterior blastema regions in case of the pERK immunostaining (FI-Blastema). When carried out an in vivo ROS/H_2_O_2_ stain, the mean intensity values were obtained from the H- and R-wound region (FI-Wound). In both cases, the background mean pixel intensity values were obtained (FI-Background; average of 3 distinct regions in the rest of the fragment, except the region at the ventral nerve cords and photoreceptors as there is a stronger pERK specific signal). The fluorescence intensity was expressed as the mean FI-Blastema or FI-Wound divided by the mean FI-Background, representing how many times the signal in the blastema or wound region is higher compared with the background.

### In vivo general reactive oxygen species (ROS) detection

The compound 5-(and-6)-carboxy-2′,7′-dichlorodihydrofluorescein diacetate (carboxy-H_2_DCFDA, Image-iT LIVE Green Reactive Oxygen Species Detection Kit, Molecular Probes; Invitrogen, I36007) was used to visualize the general in vivo production of ROS, in which fluorescent carboxy-DCF is produced through ROS oxidation after removal of the acetate groups by intracellular esterases. The ROS visualization procedure was performed on *Smed-egfr-3* RNAi knockdown animals as well as control- and MEK-inhibited animals either combined or not with the inhibition of ROS production by DPI or light therapy. Animals were exposed to carboxy-H_2_DCFDA (25 μM, 1 ml) for 1 h prior to amputation and for 1 day post RNAi. Amputated animals were again incubated in carboxy-H_2_DCFDA for 15 min before immobilization in 2% (w/v) low melting point (LMP) agarose (Invitrogen, 16520-050). Imaging of the samples was performed on 30 min, 6- and/or 24 h post amputation (MPA/HPA) using a MZ16F fluorescence stereomicroscope (Leica) combined with a ProgRes C3 camera (Jenoptik, Jena, Germany) or a Ts2-FL inverted microscope (Nikon) combined with a Ds-Fi3 color camera (Nikon). For all pictures the exact same capturing settings were used. Additionally, all experiments were also performed without the carboxy-H_2_DCFDA-stain in order to discard possible autofluorescence at the wound sites.

### In vivo H_2_O_2_ detection

The compound 2′,3′,6′,7′-Tetrahydro-12′-(4,4,5,5-tetramethyl-1,3,2-dioxaborolan-2-yl)-spiro[isobenzofuran-1(3H) or Peroxy Orange 1 (PO1, Sigma-Aldrich, SML0688) was used to specifically stain in vivo, intracellular H_2_O_2_. After initial range finding experiments, worms were incubated for 1 h in 20 μM PO1 working solution (stock- in DMSO, working solution in fresh cultivation medium) prior to (re)wounding depending on the experimental setup. After amputation, samples were followed by another 15 min incubation in the same staining solution. Next, worms were gently rinsed with medium and imaged using a fluorescent microscope (Nikon Eclipse 80i) with a Nikon DS-Ri2 digital camera. For all pictures the exact same capturing settings were used. Additionally, all experiments were also performed without the PO1-stain in order to discard possible autofluorescence at the wound sites.

### Statistical analysis

Data was analyzed using a one- or two-way ANOVA followed by a Tukey HSD post-hoc test for multiple comparisons. Normality was checked by Shapiro–Wilk, followed by a transformation of the data set (ex, 1/x, Square root and Log) if the assumptions of normality were not met. All analyses were performed with RStudio 0.98.1103 (Rstudio, Inc.) *p* values < 0.05 were considered significant.

## Data availability

The authors declare that all data supporting the findings of this study are available within the article and its Supplementary Information files or from the corresponding author upon reasonable request.

## Supplementary information

Supplementary Figures.
