# Supplementary material for: Reactive oxygen species rescue regeneration after silencing the MAPK–ERK signaling pathway in Schmidtea mediterranea
Source: Sci Rep. 2021 Jan 13;11:881. doi: 10.1038/s41598-020-79588-1 (PMC7806912; doi:10.1038/s41598-020-79588-1)
Supplement: Supplementary file 1 — Supplementary Figures. [file 41598_2020_79588_MOESM1_ESM.pdf]

SUPPLEMENTARY INFORMATION

Reactive oxygen species rescue regeneration after silencing the MAPK-ERK signaling pathway in *Schmidtea mediterranea*

V. Jaenen[1], S. Fraguas[2,3], K. Bijnens[1], M. Heleven[1], T. Artois[1], R. Romero[2], K. Smeets[1]†*, F. Cebrià[2,3]†*

[1] Centre for Environmental Sciences, Hasselt University, Diepenbeek, Belgium.

[2] Department of Genetics, Microbiology and Statistics, Faculty of Biology, University of Barcelona, Barcelona, Spain.

[3] Institute of Biomedicine of the University of Barcelona (IBUB), University of Barcelona, Barcelona, Spain.

† These authors contributed equally to this work

* Co-Corresponding authors. Emails: karen.smeets@uhasselt.be (K.S); fcebrias@ub.edu (F.C)

Francesc Cebrià

Department of Genetics, Microbiology and Statistics

Faculty of Biology

Av. Diagonal 643, 08028 Barcelona, Spain

Karen Smeets

Department of Biology and Geology

Faculty of Sciences

Agoralaan Building D, 3590 Diepenbeek, Belgium

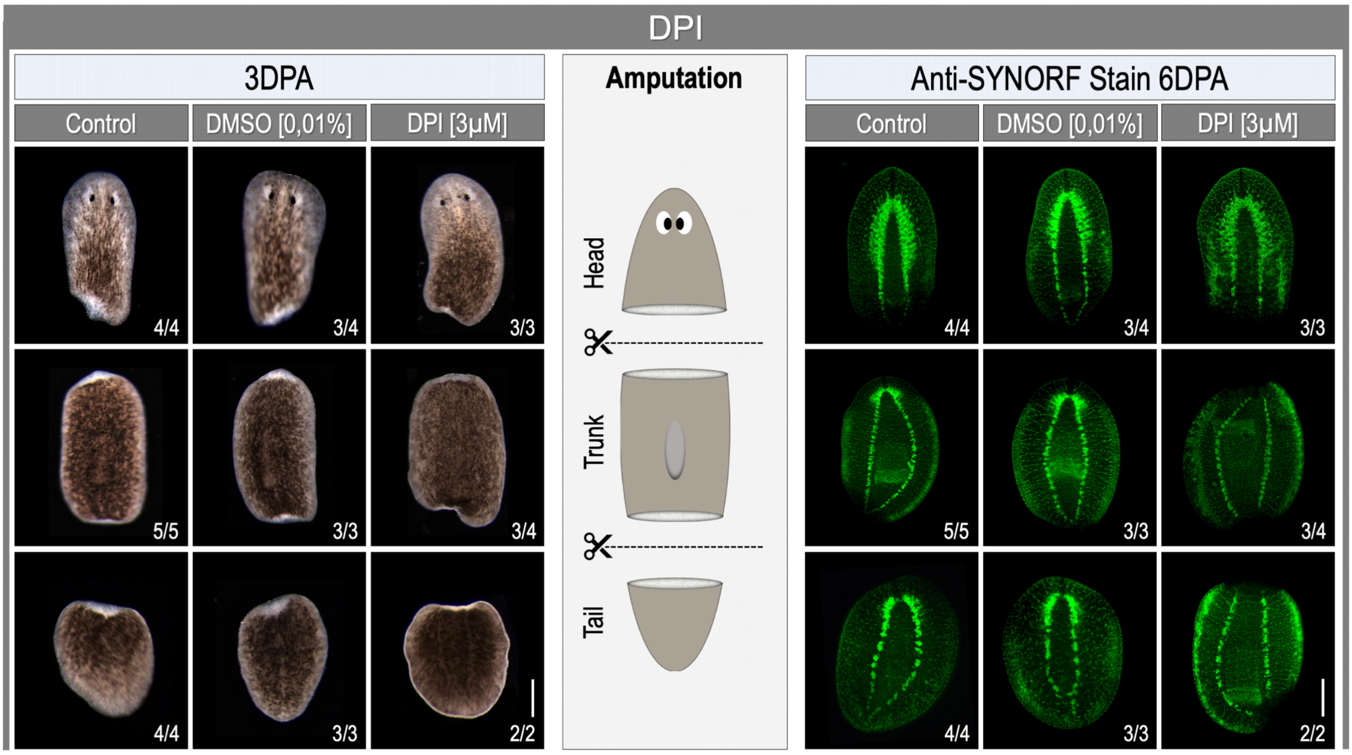

Supplementary Fig. S1: DPI mediated inhibition of ROS production impairs blastema formation and CNS regeneration. Control animals were kept in culture medium. In an additional control group, animals were exposed to 0,01% DMSO, while ROS inhibition was mediated by exposure to 3 µM DPI. In the left panels, all images were taken at 3 days post amputation (3DPA). The amputation setup is shown in the central panel. The right panels show regenerating fragments after an immunostaining with anti-SYNORF1 in order to label the central nervous system at 6 days post amputation (6DPA). Scale bar 200 µm.

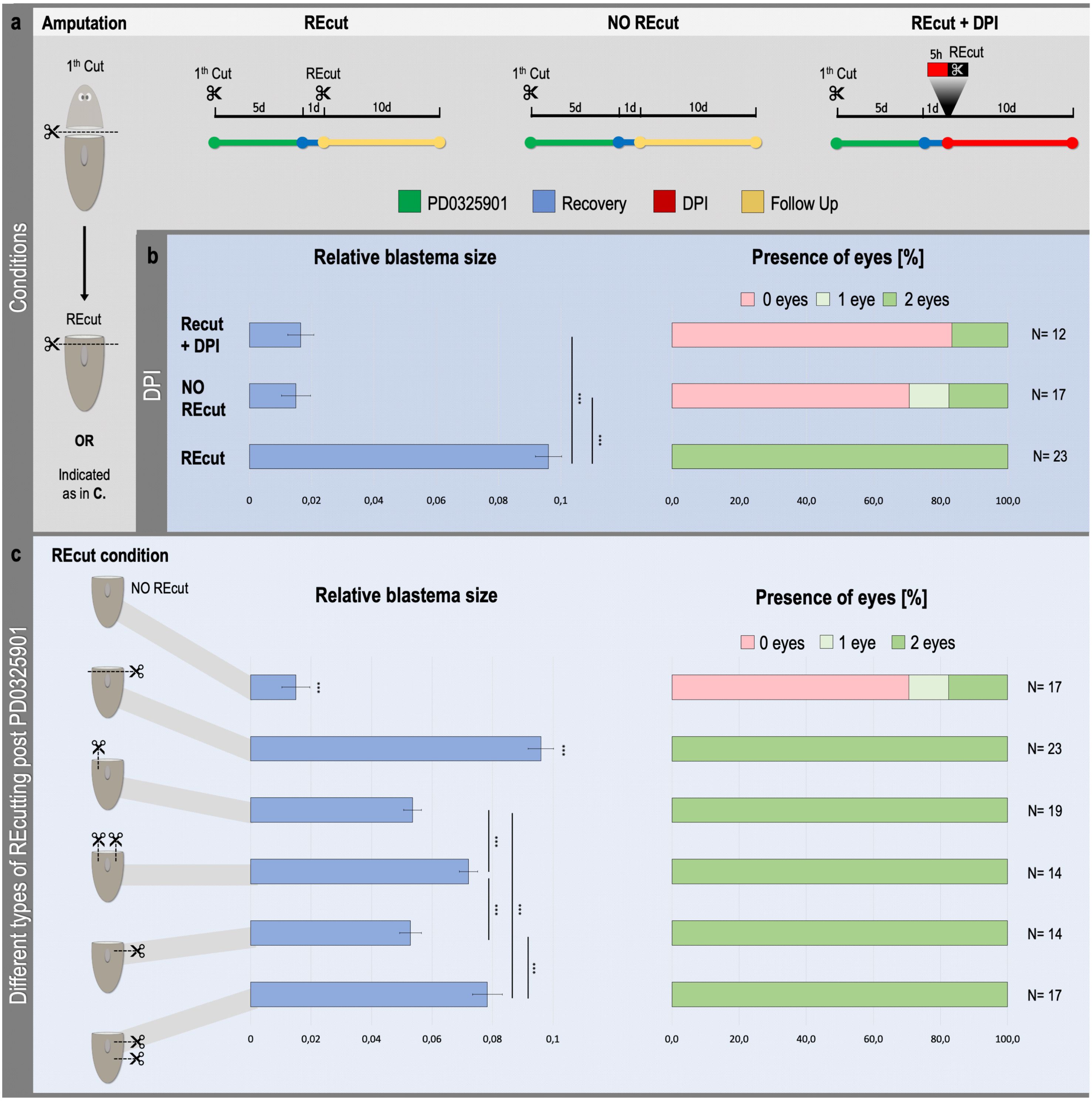

a
Conditions
Amputation
1th Cut
REcut
OR
Indicated as in C.
REcut
1th Cut
REcut
5d
1d
10d
NO REcut
REcut + DPI
5h
PD0325901
Recovery
DPI
Follow Up
b
DPI
Relative blastema size
Presence of eyes [%]
0 eyes
1 eye
2 eyes
Recut + DPI
NO REcut
REcut
N= 12
N= 17
N= 23
0
0,02
0,04
0,06
0,08
0,1
0,0
20,0
40,0
60,0
80,0
100,0
c
Different types of REcutting post PD0325901
REcut condition
NO REcut
Relative blastema size
Presence of eyes [%]
0 eyes
1 eye
2 eyes
N= 17
N= 23
N= 19
N= 14
N= 14
N= 17

Supplementary Fig. S2: DPI prevents rewounding-induced rescue of regeneration after MEK-inhibition. H-wounding in the flank rescues regeneration at the dormant R-wound site after MEK-inhibition. (**a**) For each condition indicated above, the experimental setup is displayed. Color code as follows; green: MEK inhibition by PD0325901 (10 µM,5 days), blue: recovery in fresh medium after several washes (1 day), red: treatment with DPI (3 µM, 5 hours prior REcut + during regeneration for 10 days), yellow: follow up in fresh medium after several washes (10 days). (**b**) Dormant MEK-inhibited trunk fragments were "REcut" or treated with DPI ("NO REcut + DPI"). "NO REcut" controls were neither recut or treated with DPI and were only MEK-inhibited. (**b**, left panel) Relative blastema sizes in each condition. Statistical significance is indicated by: *: $p < 0.05$; **: $p < 0.01$; ***: $p < 0.001$. (**b**, right panel) Development of the eyes. Legend as follows; green: two eyes, light green: 1 eye, red: no eyes. Results represent 2 independent experiments with 23 (REcut), 17 (NO REcut) & 12 (REcut + DPI) biological replicates. All measurements were carried out 10 days post re-wounding (REcut & REcut + DPI) or 10 days post recovery day (NO REcut). We want to emphasize the similar results between rewounding MEK-inhibited tails in the presence of DPI (Recut + DPI) and the condition without any further treatment or rewounding (NO Recut). (**c**) Dormant MEK-inhibited trunk fragments were "REcut" in different ways, left panel from the top down; NO REcut, R-wound, 1 H-wound in dormant wound site, 2 H-wounds in dormant wound site, 1 H-wound in flank below dormant wound site, 2 H-wounds in flank below dormant wound site. (**c**, middle panel) Relative blastema sizes in each condition. Statistical significance is indicated by: *: $p < 0.05$; **: $p < 0.01$; ***: $p < 0.001$. (**c**, right panel) Eye development. Legend as follows; green: two eyes, light green: 1 eye, red: no eyes. Results represent 2 independent experiments with: 17 (NO REcut), 23 (R-wound), 19 (1 H-wound in dormant wound site), 14 (2 H-wounds in dormant wound site), 14 (1 H-wound in flank below dormant wound site) and 17 (2 H-wounds in flank below dormant wound site) biological replicates. All measurements were carried out 10 days post re-wounding or 10 days post recovery day (NO REcut).

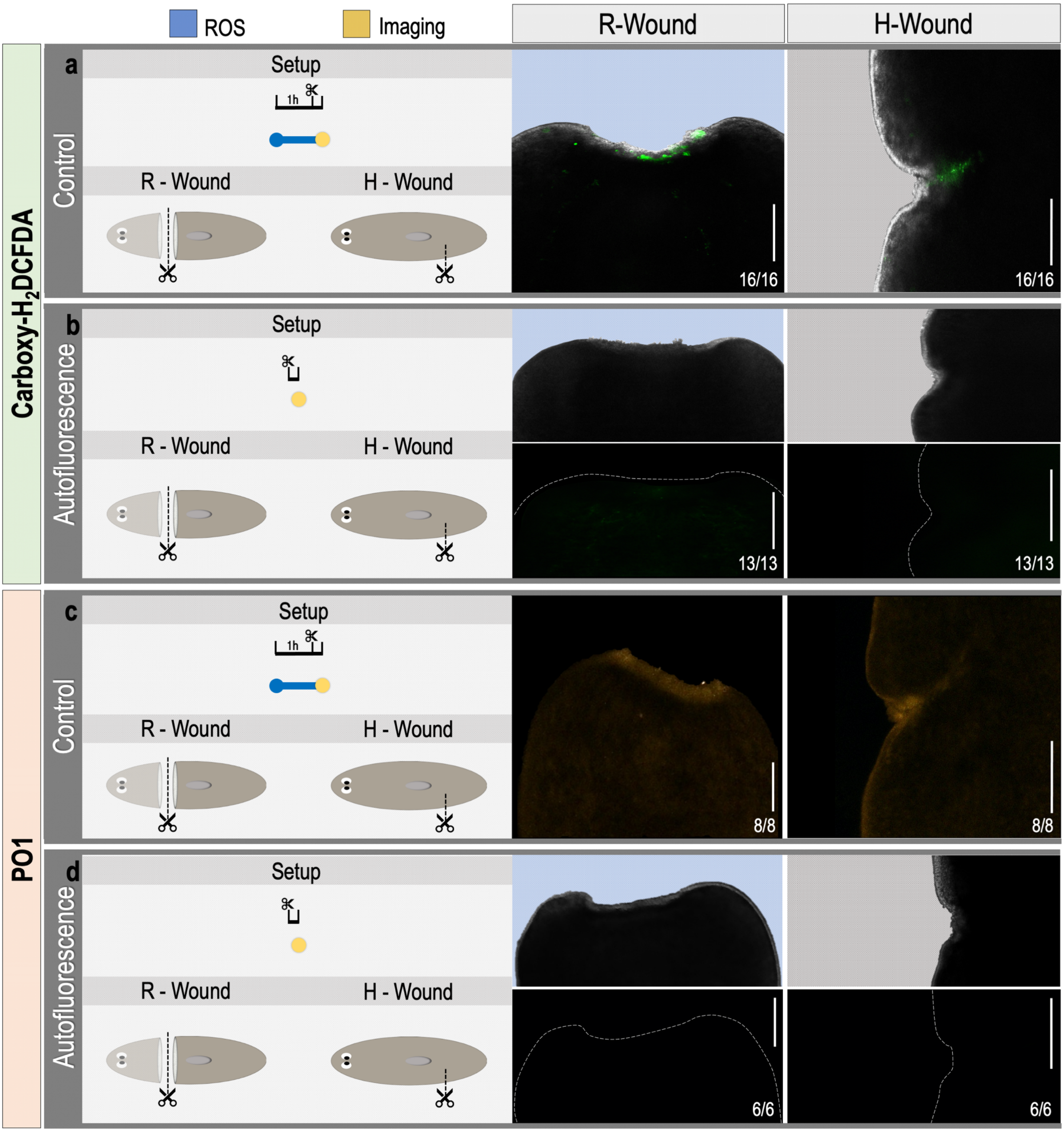
ROS
Imaging
R-Wound
H-Wound
Carboxy-H2DCFDA
a
Control
Setup
1h
R - Wound
H - Wound
16/16
16/16
b
Autofluorescence
Setup
R - Wound
H - Wound
13/13
13/13
PO1
c
Control
Setup
1h
R - Wound
H - Wound
8/8
8/8
d
Autofluorescence
Setup
R - Wound
H - Wound
6/6
6/6

Supplementary Fig. S3: The fluorescent signal at the wound site is ROS production specific and not related to autofluorescence. For each condition, the experimental setup is displayed in the left panel. Color code as follows; blue: general ROS visualization procedure using carboxy-H_2DCFDA to stain ROS or peroxy-orange 1 to specifically stain H_2O_2, yellow: imaging procedure. Amputation setup is displayed directly below. Regenerative wound (R-wound) and healing wound (H-wound) are indicated. All animals were imaged 30 minutes post amputation (MPA). A representative close-up image (merged or separated: bright field and fluorescence) of either an R-wound or H-wound is displayed on the right panel. (**a**) Control condition with general ROS visualization at the site of the R-wound (**a**, left panel) and H-wound (**a**, right panel) indicated by the green fluorescent signal, as well as (**c**) H_2O_2 visualization at the R wound (**c**, left panel) and H-wound (**c**, right panel) illustrated by the orange fluorescent signal. (**b**/**d**) No *in vivo* ROS stain is performed in order to exclude possible autofluorescence at the wound sites. In both R- (**b/d**, left panels) and H-wound (**b/d**, right panels), no autofluorescence was detected. Because of the absence of a fluorescence signal, the close-up of the wound site is shown in bright field (upper panel) and fluorescence (lower panel) separated. The dotted, white line indicates the border of the wound site. Sample numbers are indicated in the images. Scale bar 100 µm.

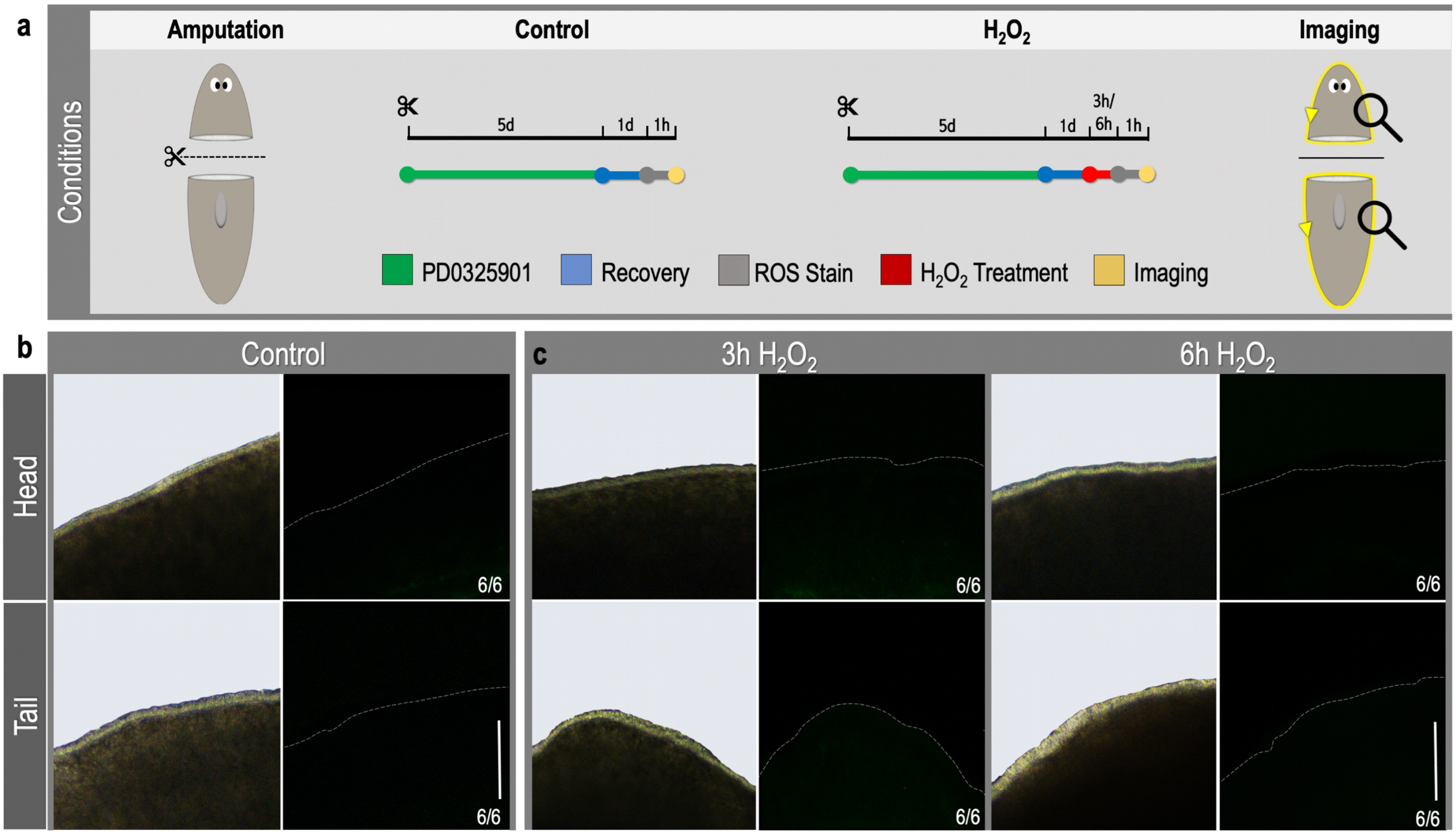

Supplementary Fig. S4: H_2O_2 treatment does not induce wounding. (**a**) For each condition, the experimental setup is displayed. Color code as follows; green: MEK inhibition by PD0325901 (10 µM, 5 days), blue: recovery in fresh medium after several washes (1 day), grey: general ROS visualization procedure using carboxy-H_2DCFDA, red: treatment with H_2O_2 (1,5 mM, 3 or 6 hours), yellow: imaging procedure. The amputation setup is displayed at the left side; amputation above the pharynx generating a head and tail fragment. H_2O_2 treated fragments were *in vivo* stained and imaged after either 3 - or 6 hours of exposure to H_2O_2. (**b**) A close-up of the epidermis is presented in bright field (left panel) and fluorescence (right panel) separated and is representable for the whole fragment. (b) control condition: MEK-inhibited fragments without any other intervention. (**c**) treatment conditions: MEK-inhibited fragments followed by 3- (**c**, left panels) or 6 hours (**c**, right panels) of H_2O_2-treatment. Sample numbers are indicated in the images. The dotted, white line indicates the border of the fragment. Scale bar 50 µm.

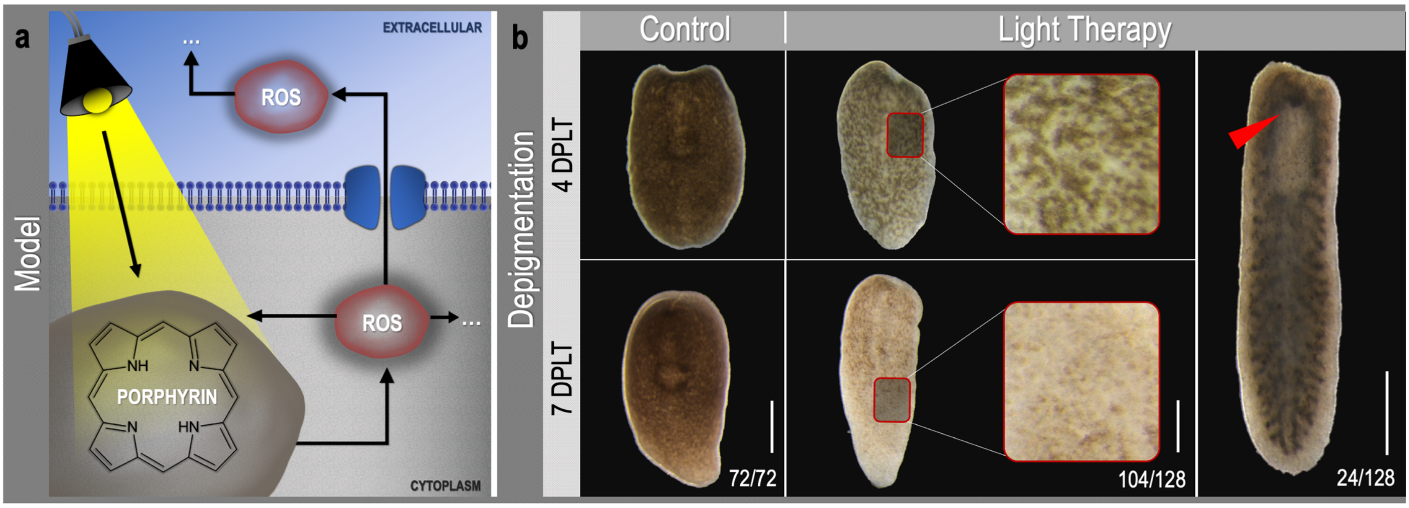

Supplementary Fig. S5: Light therapy induces a ROS-related depigmentation of the worm. a) Proposed mechanism after exposure of planarians pigment cells to visible light in which light therapy triggers porphyrin pigment-induced ROS production [36,37]. **b**) Pictures of MEK-inhibited tail fragments on 4- and 7-Days Post Light Therapy (DPLT). Controls were MEK-inhibited but not treated with light. A four times magnification of the depigmented skin is shown in the pictures with a red outline. The right panel shows the phenotype of a dark deposition in the guts induced by light therapy. Sample numbers are indicated in the images. Scale bars 100 µm.

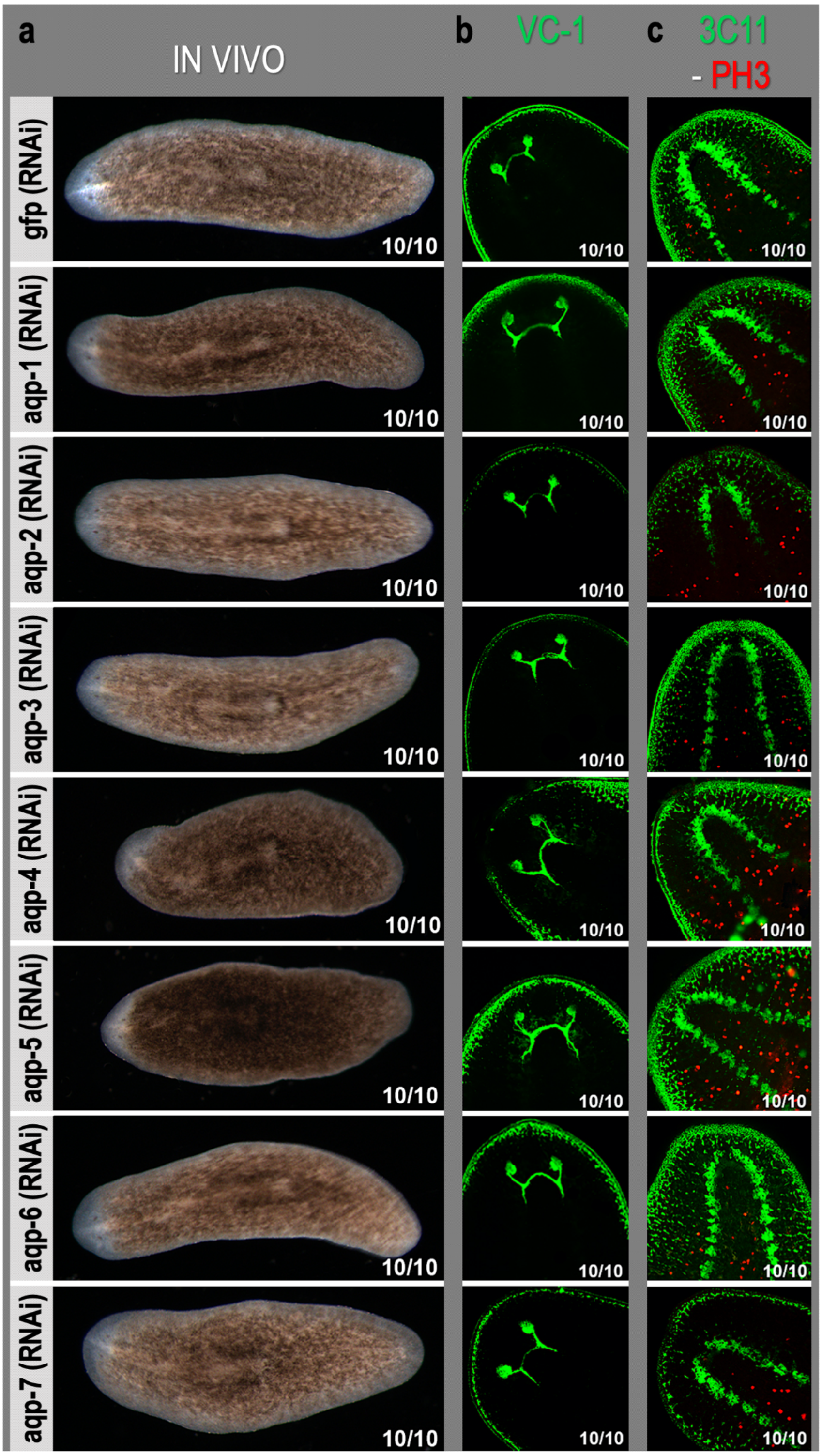

Supplementary Fig. S6: Silencing of aquaporins does not impair regeneration. Control animals were subjected to RNAi of gfp while in the treatment groups the individual aquaporins (aqp-1 to aqp-7) were knockdowned. **a**) Animals were phenotypically followed up. **b**) VC-1 immunofluorescence to label the optic chiasm formed by axons of the photosensitive cells. **c**) Whole-mount immunostaining with an anti- synapsin (3C11) antibody in order to visualize the central nervous system (green signal) in combination with labeling the mitotically active celnti-phospho-Histon-H3 antibody (pH3, red signal). Sample numbers are indicated in the images.

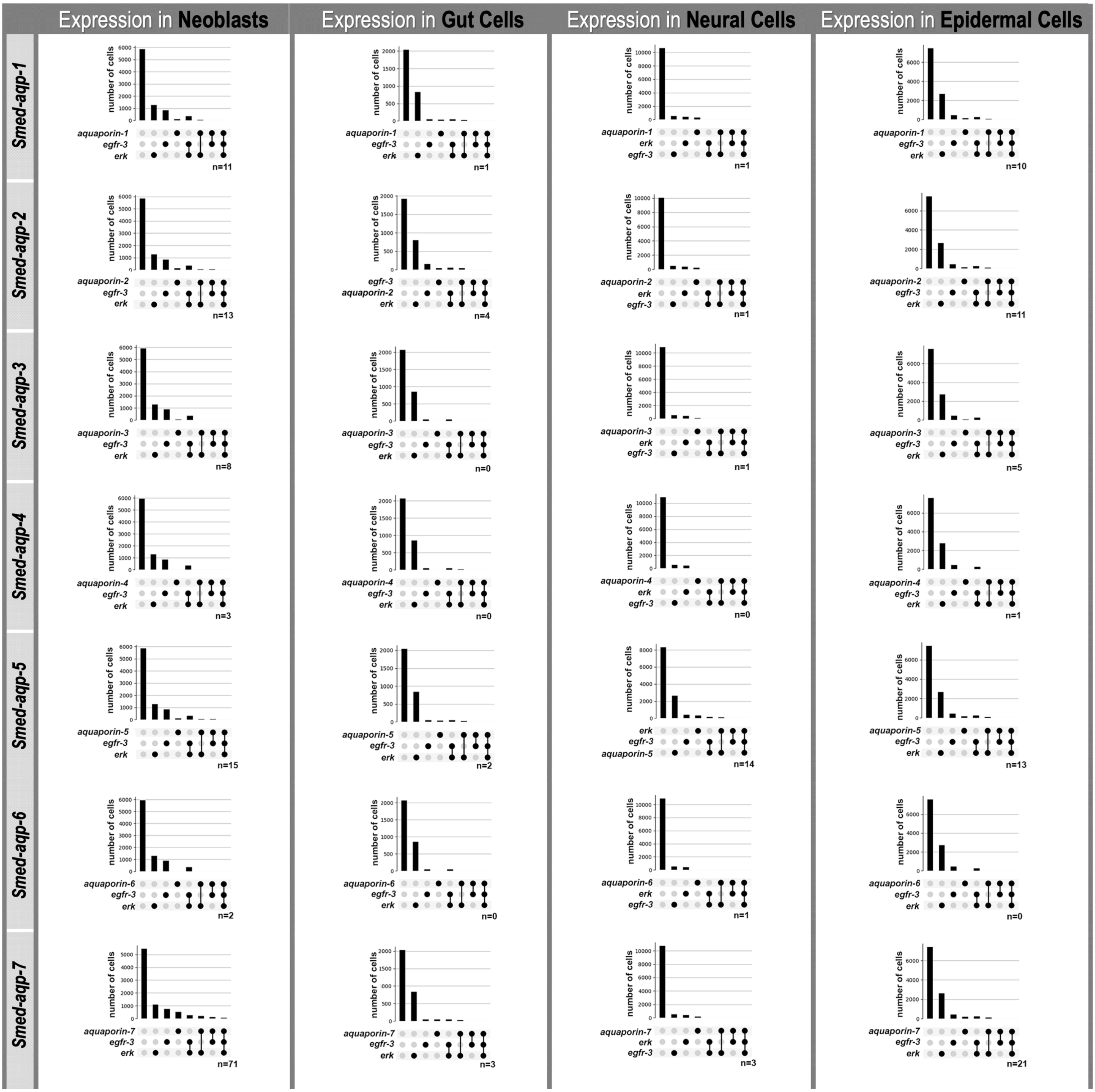
Expression in Neoblasts
Expression in Gut Cells
Expression in Neural Cells
Expression in Epidermal Cells
Smed-aqp-1
Smed-aqp-2
Smed-aqp-3
Smed-aqp-4
Smed-aqp-5
Smed-aqp-6
Smed-aqp-7
number of cells
aquaporin-1
aquaporin-2
aquaporin-3
aquaporin-4
aquaporin-5
aquaporin-6
aquaporin-7
egfr-3
erk
n=11
n=1
n=1
n=10
n=13
n=4
n=1
n=11
n=8
n=0
n=1
n=5
n=3
n=0
n=0
n=1
n=15
n=2
n=14
n=13
n=2
n=0
n=1
n=0
n=71
n=3
n=3
n=21

Supplementary Fig. S7: *Smed-aquaporin*, *Smed-erk* and *Smed-egfr-3* are co-expressed mainly in neoblasts, neurons and epidermal cells. Expression and co-expression of Smed-aquaporin (1-7), *Smed-erk* and *Smed-egfr-3* based on single cell sequencing available [41,42]. At the top of the figure the analyzed lineages are mentioned, while at the very left the particular type of aquaporin (1-7) can be found of which the co-expression has been verified. For each of the lineages and specific type of aquaporin, data is visualized in an individual graph in which the first column represents the number of cells of that particular lineage in which neither *aquaporin-(1-7)*, *Smed-egfr-3* nor *Smed-erk* are expressed. The second, third and fourth column indicate the number of cells expressing *Smed-erk, Smed-egfr-3* and *Smed-aquaporin-(1-7)* respectively. The fifth column indicates the number of cells in which *Smed-egfr-3* and *Smed-erk* are co-expressed. The sixth column indicates the number of cells in which *Smed-aquaporin-(1-7)* and *Smed-erk* are co-expressed. The seventh column indicates the number of cells in which *Smed-aquaporin-(1-7)* and *Smed-egfr-3* are co-expressed. Finally, the eighth column indicates the number of cells in which *Smed-aquaporin-(1-7)*, *Smed-egfr-3* and *Smed-erk* are co-expressed.
